# Generic design principles for antibody-based tumour necrosis factor (TNF) receptor 2 (TNFR2) agonists with FcγR-independent agonism

**DOI:** 10.7150/thno.84404

**Published:** 2024-01-01

**Authors:** Mohamed A. Anany, Stefanie Haack, Isabell Lang, Julia Dahlhoff, Juan Gamboa Vargas, Tim Steinfatt, Lea Päckert, Daniela Weisenberger, Olena Zaitseva, Juliane Medler, Kirstin Kucka, Tengyu Zhang, Tom Van Belle, Luc van Rompaey, Andreas Beilhack, Harald Wajant

**Affiliations:** 1 Division of Molecular Internal Medicine, Department of Internal Medicine II, University Hospital Würzburg, Würzburg, Germany.; 2 Department of Microbial Biotechnology, Institute of Biotechnology, National Research Center, Dokki, Giza, Egypt.; 3 Department of Internal Medicine II, Interdisciplinary Center for Clinical Research (IZKF) laboratory Würzburg, Center for Experimental Molecular Medicine, University Hospital Würzburg, Würzburg, Germany.; 4 Dualyx NV, 9052 Zwijnaarde, Belgium.

## Abstract

**Background:** Selective TNFR2 activation can be used to treat immune pathologies by activating and expanding regulatory T-cells (Tregs) but may also restore anti-tumour immunity by co-stimulating CD8^+^ T-cells. Oligomerized TNFR2-specific TNF mutants or anti-TNFR2 antibodies can activate TNFR2 but suffer either from poor production and pharmacokinetics or in the case of anti-TNFR2 antibodies typically from the need of FcγR binding to elicit maximal agonistic activity.

**Methods:** To identify the major factor(s) determining FcγR-independent agonism of anti-TNFR2 antibodies, we systematically investigated a comprehensive panel of anti-TNFR2 antibodies and antibody-based constructs differing in the characteristics of their TNFR2 binding domains but also in the number and positioning of the latter.

**Results:** We identified the domain architecture of the constructs as the pivotal factor enabling FcγR-independent, thus intrinsic TNFR2-agonism. Anti-TNFR2 antibody formats with either TNFR2 binding sites on opposing sites of the antibody scaffold or six or more TNFR2 binding sites in similar orientation regularly showed strong FcγR-independent agonism. The affinity of the TNFR2 binding domain and the epitope recognized in TNFR2, however, were found to be of only secondary importance for agonistic activity.

**Conclusion:** Generic design principles enable the generation of highly active bona fide TNFR2 agonists from nearly any TNFR2-specific antibody.

## Introduction

Tumour necrosis factor (TNF) inhibitors reached prominence as potent biologicals to treat a wide variety of inflammatory diseases. TNF acts via two receptors which are typical members of the TNF receptor superfamily (TNFRSF) 1. TNF receptor 1 (TNFR1) belongs to the death receptor subgroup of the TNFRSF, is expressed by almost any type of cell and was early on recognized as a strong pro-inflammatory receptor. Indeed, the overwhelming clinical success of TNF blockers in the treatment of autoimmune diseases reflects the inhibition of TNFR1 activation 2-4. The for a long time neglected TNFR2 belongs to the TNF receptor-associated factor (TRAF)-interacting subgroup of the TNFRSF and is mainly expressed by myeloid cells, certain types of T- and B-cells, neurons and endothelial and epithelial cells 5. Although, TNFR2 exerts pro-inflammatory effects, e.g. co-stimulation of cytotoxic T-cells, it also elicits strong and manifold anti-inflammatory activities, and promotes tissue homeostasis and regeneration. TNFR2 stimulates Tregs, regulatory B-cells, myeloid-derived suppressor cells, mesenchymal stem cells and endothelial progenitor cells 5-9. Furthermore, TNFR2 activation exerts protective activities on various cell types, including oligodendrocytes, cardiomyocytes, and keratinocytes 5. TNFR2 agonists attract therefore currently considerable interest for the treatment of autoimmune diseases, neurodegenerative diseases and even cancer 2-6.

TNFR2 activation depends on secondary clustering of two or more liganded TNFR2 trimers 5,10. Indeed, membrane-bound TNF (memTNF), soluble TNF and LTα are ligands of the TNF superfamily (TNFSF) and bind three molecules of TNFR2 resulting initially in no or only limited TNFR2 activation 10. Similar to several other receptors of the TNFRSF (TNFRs), TNFR2 molecules auto-aggregate only with low affinity 10. In the case of memTNF-engaged TNFR2 molecules, this weak auto-affinity suffices in the cell-to-cell contact zone of memTNF- and TNFR2-expressing cells to promote spontaneous clustering of trimeric liganded receptor complexes and subsequent receptor activation. Importantly, soluble TNF liganded trimeric TNFR2 complexes remain largely inactive 11,12 presumably by insufficient receptor-mediated clustering**.** However, clustering of soluble TNF liganded TNFR2 trimers and subsequent receptor activation can be invoked by the enforced connection of two or more soluble ligand trimers, e.g. by ligand cross-linking antibodies or genetic fusion of soluble TNF with appropriate oligomerizing domains 12,13. Similar principles apply to anti-TNFR2 antibodies. Conventional TNFR2-specific IgG molecules are bivalent and thus do not form hexameric receptor complexes. However, antibody crosslinking forces the IgG-bound TNFR2 dimers together into clusters and in this spatial context receptor activation can again take place 10. In the cell-to-cell contact zone between FcγR-expressing and TNFR2-expressing cells super-high concentrations of TNFR2 dimers bound to FcγR-associated antibodies can be reached so that TNFR2 complexes may again auto-aggregate via their low intrinsic auto-affinity resulting in strong activation 14. Previously, we and others have developed soluble TNF-based agonists that allow selective stimulation of TNFR2 due to their oligomeric nature and mutations conferring selectivity for TNFR2 13,15,16. Yet, the translational development of such biologicals is challenging. Therefore, agonistic TNFR2-specific antibodies would still be the reagent of choice for therapeutic TNFR2 activation. However, since conventional TNFR2 antibodies typically require FcγR binding to be agonistic or to show maximal agonism (**Supplemental Table S1**), crucial FcγR-related limitations have to be considered. First, FcγR activation, which is triggered by the bound anti-TNFR2 antibody, may counteract or even negate the anticipated effects of TNFR2 activation. Second, expression levels of freely available FcγR on immune cells per se, but also the availability of the immune cells in the vicinity of TNFR2-expressing target cells will clearly limit the activity that can be reached with FcγR-dependent agonistic TNFR2-specific antibodies. Last but not least, the competition for TNFR2 binding between highly agonistic FcγR-bound anti-TNFR2 molecules and non/poorly-agonistic “free” anti-TNFR2 molecules will unavoidably dampen the agonistic TNFR2 net-response, too. Thus, agonistic activity of anti-TNFR2 antibody formats depending on FcγR-engagement may bare the risk of unpredictable and even potentially adverse outcomes not related to TNFR2 signaling.

In this study, we identified two independent but combinable strategies to overcome the limitations of conventional anti-TNFR2 antibodies with FcγR-dependent agonism: First, by increasing the valency of unidirectional aligned TNFR2-binding sites and second by empowering anti-TNFR2 antibodies with cell-cell-connecting capacity. Notably, following these design principles, practically any TNFR2-specific antibody, even antagonistic ones, become suitable to generate potent TNFR2-specific agonists.

## Results

### Agonism of anti-TNFR2 antibody C4 variants with TNFR2 binding domains on opposing sides of the molecule

**Figure 1:**
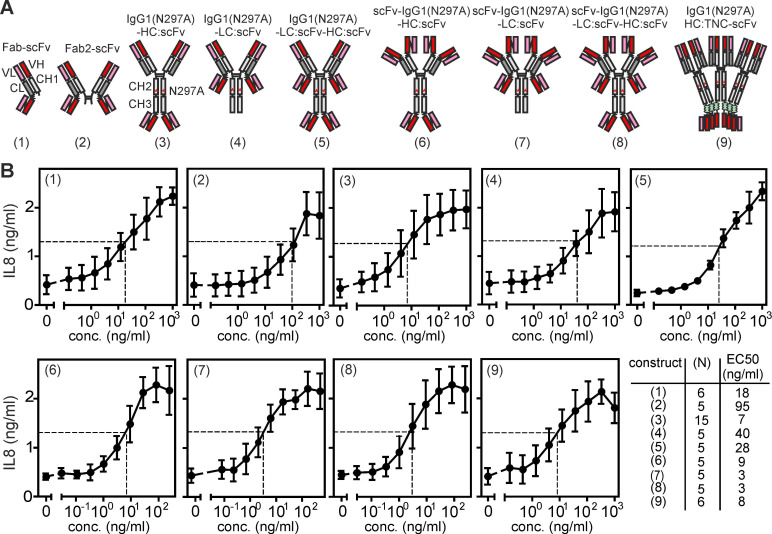
** Antibody variants with N- and C-terminal TNFR2 binding sites harness strong TNFR2 agonism.** (**A**) Domain architecture of antibody variants with TNFR2 binding domains on opposing sides of the molecule. (**B**) Intrinsic agonism of C4 antibody variants with N- and C-terminal TNFR2 binding sites. HT1080-TNFR2 cells were stimulated with the indicated concentrations of the various C4 constructs and the next day IL8 production was quantified by ELISA. The half maximal TNFR2 response level induced by the highly agonistic TNFR2-specific TNF variant TNC-sc(mu)TNF80 is indicated by a dotted line. Shown are averaged data of 5-15 independent experiments. The number of experiments for each construct and their EC_50_ values are listed in the table.

In view of the cell-to-cell contact associated mode of TNFR2 activation by memTNF and FcγR-bound anti-TNFR2 IgG antibodies, we predicted that antibody variants with the capability to interact simultaneously with two TNFR2-expressing cells have an intrinsic FcγR-independent agonistic activity and thus act as bona fide TNFR2-specific agonists. To experimentally test this hypothesis, we engineered and analyzed a panel of variants of the antagonistic anti-TNFR2 antibody C4 (**Supplemental Figure S1,** ref. 14) placing TNFR2 binding sites on opposing positions of an IgG1 scaffold. To largely rule out FcγR-independent agonism, we introduced the N297A mutation into the IgG1 antibody scaffold to prevent/minimize interaction with FcγRs. By fusing a scFv:C4 domain to the C-terminus of the light chain of C4 Fab we generated a simple antibody format with two oppositely oriented TNFR2 binding domains (**Figure 1A**, variant (1)). Furthermore, we created tetravalent constructs with oppositely oriented TNFR2 binding sites by fusing the scFv:C4 domain to the C-terminus of the heavy or light chain of the parental C4-IgG1(N297A) antibody (**Figure 1A**, variants (3) and (4)) or to the light chain of C4-Fab_2_ (**Figure 1A**, variant (2)). Next we engineered C4 variants with six oppositely oriented TNFR2 binding sites by fusing scFv:C4 domains to the C-terminus of the light and heavy chain of C4-IgG1(N297A) (**Figure 1A**, variant (5)) and by fusing the scFv:C4 domain to the C-terminus of either the light or heavy chain of an IgG1(N297A) scaffold in which we had replaced the variable domains with scFv:C4 domains (**Figure 1A**, variant (6) and (7)). Furthermore, we designed an octameric C4 variant with oppositely oriented binding sites by connecting the C-terminus of the heavy and light chain of the tetravalent scFv:C4-IgG1(N297A) scaffold with the scFv:C4 domain (**Figure 1A**, variant (8)). Finally, we engineered a dodecameric C4 variant with oppositely oriented binding sites by fusing the TNC trimerization domain and a scFv:C4 domain to the C-terminus of the C4-IgG1(N297A) heavy chain (**Figure 1A**, variant (9)).

All C4 variants were transiently produced in HEK293 cells and contained a N-terminal Flag tag in their light and heavy chain. The use of Flag-tagged antibody variants allowed the quantification of the structurally different molecules in cell culture supernatants by Western blotting and use of a Flag-tagged protein standard. The Flag tag enabled it also to purify the various proteins using the same and gentle method of Flag affinity purification, without the need of an acidic pH elution step, which may differently affect Fab and scFv domains. Western blot analysis showed that the Fab/scFv chimeric construct types (1) to (4) and (9) of C4, in which a scFv domain has been fused to only one of the two chains, were comparably well expressed as the parental antibody C4-IgG1(N297A) with 10-60 μg/ml and showed a fair chain balance in the supernatant and after purification (**Supplemental Figure S2**). In contrast, all scFv:C4 domain-only variants (C4-(6) to C4-(8), in which the variable domains have been replaced by the scFv:C4 domain showed significant lower expression levels of approximately 4 to 17 μg/ml (**Supplemental Figure S2**). C4-(5), in which both antibody chains have been fused to a scFv domain, showed very low production and poor chain balance. From experiments with the parental anti-TNFR2 antibody C4 with and without Flag tagging, we have no evidence that the Flag tag affect TNFR2 binding (**Supplemental Figure S3**). To determine the ability of the various C4 constructs to engage TNFR2 signaling we analyzed their capacity to stimulate IL8 production in HT1080-TNFR2 cells. In this cell line, TNFR2 activates the classical NFκB pathway, which controls IL8 expression. In contrast to the parental (blocking) C4-IgG1(N297A) antibody, all variants with oppositely oriented TNFR2 bindings sites induced IL8 production with EC_50_ values between 3 - 95 ng/ml (**Figure 1B**). The maximum responses reached with the various variants were comparable to those obtained with a previously published, highly active ligand-based nonameric TNFR2-specific TNF variant STAR2 (TNC-sc(mu)TNF80) 16. Next, we analyzed the agonism of C4-(1) to C4-(9) antibody constructs utilizing Kym-1 cells. In this cell line, TNFR2 activation induces cell death by stimulation of endogenous TNF production and concomitant depletion of cytoplasmic anti-apoptotic complexes of TRAF2 and cIAP1 or cIAP2 17,18. The parental C4-IgG1(N297A) could not trigger cell death in Kym-1 cells. However, the C4 variants with oppositely oriented TNFR2 binding sites of type (3) to (9) triggered similar cell death as the STAR2/TNC-sc(mu)TNF80 benchmark. Yet, the type (1) and (2) constructs of C4 could not trigger Kym-1 killing (**Supplemental Figure S4A**). TNFR2-mediated depletion of TRAF-cIAP1/2 complexes furthermore promotes p100 to p52 processing, which is the biochemical hallmark of the alternative NFκB pathway 13. Therefore, we also tested a subset of the C4 variants for their ability to promote p100 processing in Kym-1 cells. Parental C4-IgG1(N297A) triggered no p100 processing while the investigated C4 variants (3), (4) and (5), similarly to STAR2 (TNC-sc(mu)TNF80), triggered p100 expression and p100 processing to p52 (**Supplemental Figure S4B**).

### Agonism of anti-TNFR2 antibody C4 variants with parallel oriented TNFR2 binding sites

**Figure 2:**
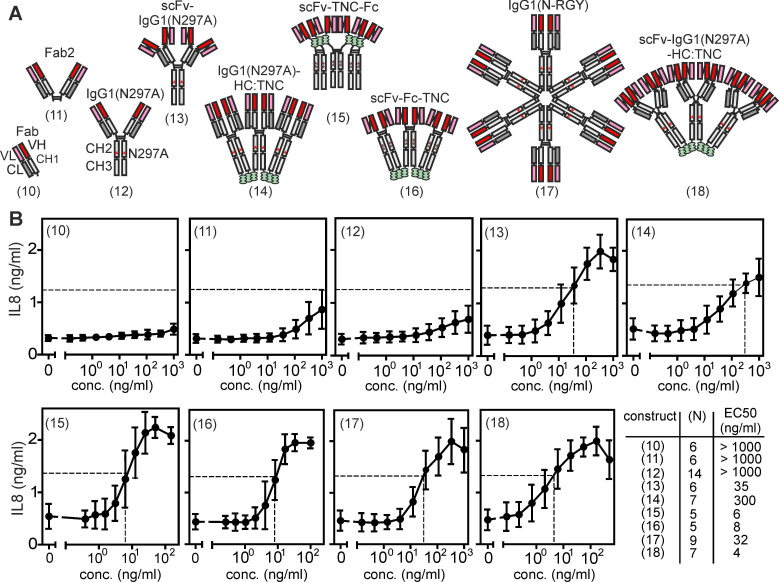
** Antibody variants with six or more unidirectional oriented N-terminal TNFR2 binding sites are intrinsically strong agonists.** (**A**) Antibody constructs with different numbers of unidirectional oriented N-terminal TNFR2 binding sites. (**B**) Intrinsic agonism of C4 antibody variants with unidirectional oriented N-terminal TNFR2 binding sites. HT1080-TNFR2 cells were stimulated with the indicated C4 variants overnight and IL8 production was quantified by ELISA. The half maximal TNFR2 response level induced by TNC-sc(mu)TNF80 is indicated by a dotted line. Shown are averaged data of 5 - 14 independent experiments. The number of experiments for each construct and their EC_50_ values are listed.

It is very well established that IgG antibodies targeting receptors of the TNFRSF, for example 41BB, CD27, CD40, CD95, DR4/TRAILR1, DR5/TRAILR2 but also TNFR2, frequently acquire strong agonistic activity upon crosslinking with protein A or secondary antibodies 19. Likewise, strong FcγR-independent intrinsic agonism has been demonstrated for pentameric antibodies, namely IgM antibodies, e.g. targeting DR4/TRAILR1 or DR5/TRAILR2 19-21. These findings suggest that avidity and/or the number of unidirectional oriented receptor binding sites can be of crucial relevance for the agonism of anti-TNFR2 antibodies. To evaluate the relevance of valency of unidirectionally oriented TNFR2 binding sites for the FcγR-independent agonism of anti-TNFR2 antibodies, we generated a second panel of variants of the anti-TNFR2 antibody C4 with one to up to 12 unidirectional oriented N-terminal TNFR2 binding sites (**Figure 2A**). First, to obtain a monovalent variant of C4, we expressed it with a truncated heavy chain resulting in a Fab fragment (**Figure 2A**, variant (10)). Second, to generate bivalent variants with unidirectional TNFR2 binding sites we expressed the conventional IgG1 molecule or a truncated heavy chain resulting in a Fab_2_ fragment (**Figure 2A**, variants (11) and (12)). Third, to create a tetravalent variant with four parallel oriented TNFR2 binding sites, we replaced the variable domains of the heavy and light chain of C4 with scFv:C4 domains (**Figure 2A**, variant (13)). Fourth, to generate hexavalent variants with similarly oriented TNFR2 binding sites we fused the scFv:C4 domain by genetic engineering to the N-terminus of fusion proteins consisting of the Fc dimerization domain and a tenascin-C (TNC)-derived trimerization domain (**Figure 2A**, variant (15) and (16)) or by connecting C4 at the C-terminus of the heavy chain of C4 with the TNC trimerization domain (**Figure 2A**, variant (14)). Fifth, to obtain a dodecavalent C4 variant we introduced mutations into C4-IgG1(N297A) promoting its hexamerization 22 or fused a TNC trimerization domain to the C-terminus to the heavy chain of construct (13) (**Figure 2A**, constructs (17) and (18)). While the constructs with conventional Fab domains were all expressed with 25 - 40 μg/ml, the expression of the scFv:C4 domain-based variants was again more variable but also reached expression levels > 10 μg/ml (**Supplemental Figure S2**). To test whether C4 antibody constructs with unidirectional oriented TNFR2 binding domains can engage TNFR2 signaling, we again determined IL8 induction in HT1080-TNFR2 cells. In contrast to the bivalent construct C4-(1) with two opposing binding sites, the bivalent variants (11) and (12), with two unidirectional oriented binding sites, displayed no relevant agonistic activity (**Figure 2B**). However, all C4 variants with 4 or more unidirectional oriented TNFR2 binding sites efficiently induced IL8 production (**Figure 2B**). The IgG1 fusion proteins C4-(14) and C4-(17) comprising 6 or 12 Fab domains induced half-maximal IL8 production with EC_50_ values of 300 and 32 ng/ml and the IgG1- and Fc-TNC-variants with scFv:C4 domains showed even a bit lower EC_50_ values of app. 4 - 35 ng/ml (**Figure 2B**). In view of the proposed need of at least two trimeric receptor complexes for engagement of robust TNFR2 signaling, the excellent activity of scFv:C4-IgG1(N297A) with its only four unidirectionally oriented binding sites was unexpected and first appeared counterintuitive. However, the scFv domains connected N-terminally to the CH1 and CL domains of the antibody by peptide linkers are “individually” movable and do not form a rigid composite binding site like the VH and VL domains of a conventional antibody. Therefore, it appears plausible that there is enough flexibility and freedom in the spatial orientation of the scFv:C4 domains in scFv:C4-IgG1(N297A), but also the other constructs with N-terminal scFv variants, to allow binding to two different TNFR2^+^ cells and to trigger spontaneous clustering of TNFR2-construct complexes in the cell-to-cell contact zone.

The ability of the C4 variants with unidirectional oriented TNFR2 binding domains to kill Kym-1 cells and to trigger p100 processing substantially differed from their ability to promote IL8 production. The bivalent C4-IgG1(N297A) and C4-Fab_2_ variants with unidirectional oriented binding sites showed again no agonistic activity (**Supplemental Figure S5A,B**). However, the unidirectional oligovalent Fab variants C4-(14) and C4-(17), despite triggering IL8 production, largely failed to kill Kym-1 cells and to promote p100 processing, while the unidirectional oligovalent scFv:C4 constructs again potently triggered these effects (**Supplemental Figure S5A,B**).

### Antibody formats (3), (13), (14) and (17) convert poorly active bivalent anti-TNFR2 antibodies into strong agonists

**Figure 3:**
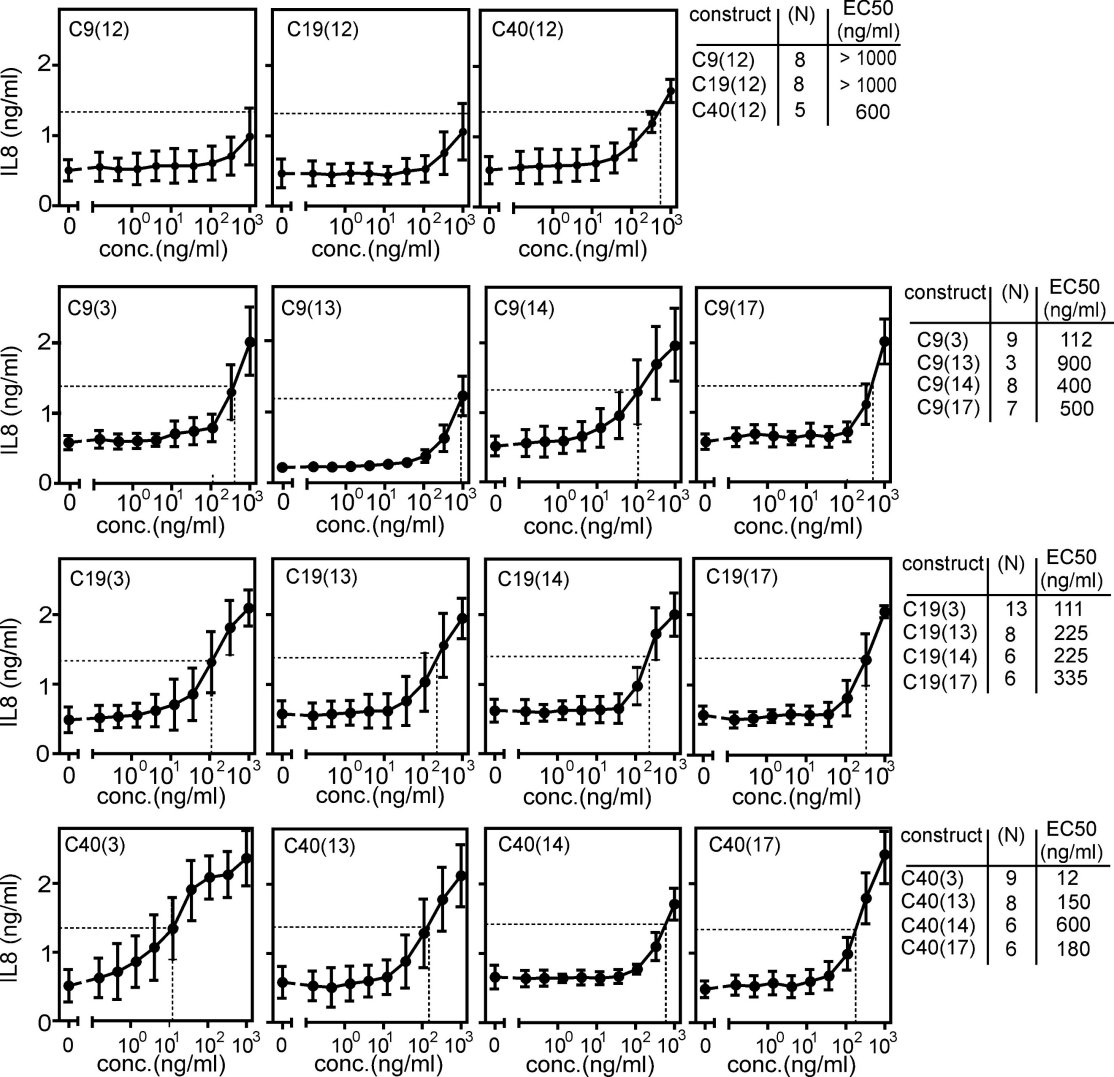
** Intrinsic agonism of format (3), (13), (14) and (17) variants of the anti-TNFR2-antibodies C9, C19 and C40.** HT1080-TNFR2 cells were stimulated with the indicated anti-TNFR2 antibody variants overnight and IL8 production was quantified by ELISA. The half maximal TNFR2 response level induced by TNC-sc(mu)TNF80 is indicated by a dotted line. Averaged data of 3 - 13 independent experiments are shown.

The functional analyses of the two panels of C4 variants with TNFR2 binding domains suggested that agonistic anti-TNFR2 antibodies variants either require oppositely oriented binding sides or six or more unidirectional oriented binding sites. To prove the general validity of this idea, we generated and analyzed variants of format (3), (14) and (17) of the anti-TNFR2 antibodies C9, C19 and C40. While C4 recognized the CRD3 of TNFR2, C9, C19 and C40 recognized CRD4, CRD1 and CRD2 of TNFR2 14. Similar to C4, the anti-TNFR2 antibody C9 blocks ligand binding (**Supplemental Figure S6A**). In contrast, the anti-TNFR2 antibodies C19 and C40 do not block ligand binding (**Supplemental Figure S6A**). Furthermore, we included the variants of these antibodies in format (13). The parental conventional IgG1(N297A) variants of the antibodies C9, C19 and C40, similarly to C4, were largely inactive as predicted. The variants (3), (13), (14) and (17) of these antibodies, on the other hand, induced robust IL8 production with EC_50_ values between 12 and 900 ng/ml (**Figure 3**). Noteworthy, however, the cell death-inducing activity of these antibody variants on Kym-1 cells varied considerably and reached from 70 - 100 ng/ml for most of the scFv/Fab construct types to poor activity > 1000 ng/ml or even inactivity for the construct type (13) and the oligomerized IgG1 variants (14) and (17) (**Supplemental Figure S6B**).

In view of the strong evidence for receptor clustering as the key factor driving TNFR2 activation, it appeared possible that biparatopic anti-TNFR2 antibody variants have a higher ability to oligomerize and thus a higher TNFR2-stimulating activity. Therefore, we generated biparatopic variants of format (3) and (13). While the biparatopic antibodies of structure (3) were regularly produced with sufficient efficacy, approximately half of the possible biparatopic variants of structure (13) showed poor expression of the heavy chain and, therefore, were not analyzed. The biparatopic constructs of type 3 were comparably active as the monoparatopic molecules but showed no enhanced activity (**Supplemental Figure S7**).

**Figure 4:**
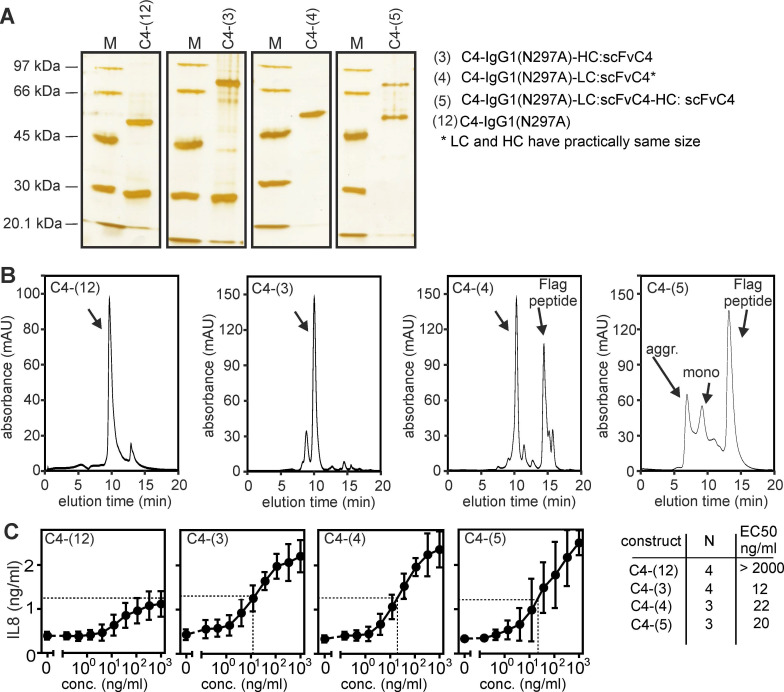
** Biochemical analysis of purified anti-TNFR2 C4 fusion proteins.** (**A**) The indicated purified proteins along with marker proteins (M) of the indicated molecular weight were separated by SDS-PAGE and visualized by silver staining. (**B**) Purified proteins shown in A were analyzed by gel filtration. (**C**) IL8-inducing activity was controlled by stimulation experiments with HT1080-TNFR2 cells.

A trivial explanation for the TNFR2-stimulating activity of an anti-TNFR2 construct could be that the molecules auto-aggregate and thus mimic conventional bivalent antibodies crosslinked by protein G or secondary antibodies. Therefore, we purified several of the anti-TNFR2 C4 variants by anti-Flag affinity chromatography (**Figure 4A**) and analyzed the assembly of the molecules by gel filtration analysis (**Figure 4B**). The majority of the purified proteins eluted according to the expected size for non-aggregated molecules and showed no or only a limited fraction of aggregates. In particular, the purified proteins displayed a comparable TNFR2-stimulating activity as the non-purified proteins before (compare **Figure 4C** with **Figures 1B** and **2B**).

**Figure 5:**
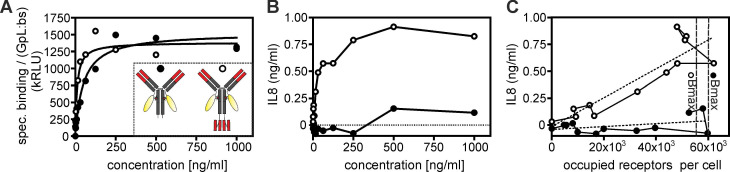
** Tetravalent mAb occupied TNFR2 molecules trigger much stronger IL8 induction than TNFR2 molecules occupied by the conventional bivalent mAb variant.** (**A,B**) TNFR2-negative Hela cells and HeLa-TNFR2 cells were pairwise stimulated for 8 hours with the indicated concentrations of the GpL fusion proteins of C4-IgG1(N297A) and C4-(3) (scheme see inset). Supernatants of HeLa-TNFR2 were then analyzed for TNFR2-induced IL8 production (B) and repeatedly washed cells (HeLa, and HeLa-TNFR2) were analyzed for cell-associated GpL activity. Specific binding to TNFR2, shown in (A) were calculated by subtraction of the unspecific binding values obtained from the HeLa cells from the total binding values derived of the HeLa-TNFR2 cells. Please note, luciferase activity was normalized according to the ratio of the number of GpL reporter domains to the number of TNFR2-binding domains within the two C4 variants (1 versus 0.5). (**C**) IL8 production by the C4 variants were directly plotted as a function of their specific binding. The latter was transformed into “occupied receptors per cells” by the help of the number of cells in the assay and the measured specific activity (RLU/molecule) of the GpL constructs. Maximum binding of the two constructs obtained from A is indicated by dashed vertical lines. The dotted lines indicate linear regression of the IL8 production as a function of receptor occupancy.

The higher avidity of the oligovalent anti-TNFR2 variants could lead to an increase in the apparent affinity opening the possibility that increased TNFR2 occupancy explains their superior agonism. Indeed, using C4-IgG(N297A) and C4-(3) variants with a *Gaussia princeps* luciferase (GpL) reporter domain (**Figure 5A**), cellular binding studies revealed that C4-(3) has an approximately 4-fold higher apparent affinity than C4-IgG(N297A) (**Supplemental Figure S8** and **Figure 5A**). To directly compare the intrinsic TNFR2-stimulating capabilities of the GpL-tagged variants of C4-IgG(N297A) and C4-(3), we determined IL8 production, thus TNFR2 activity, and TNFR2 occupancy directly from the same samples (**Figure 5A,B**). Plotting IL8 production as a function of the number of occupied TNFR2 molecules revealed that the tetravalent C4-(3) variant has a considerably higher intrinsic TNFR2-stimulating activity compared to C4-IgG1(N297A) (**Figure 5C**). Indeed, while even the complete occupation of the approximately 60.000 TNFR2 molecules per cell with C4-IgG1(N297A)-LC:GpL triggered a barely detectable IL8 response, a few thousand TNFR2-bound GpL-tagged C4-(3) molecules sufficed to robustly increase IL8 production (**Figure 5C**). Thus, the superior activity of C4-(3) is not (or only marginally) related to enhanced TNFR2 binding and instead represents a novel molecule-intrinsic quality for TNFR2 stimulation, which is not achievable with free (thus not FcγR-bound) bivalent C4 molecules.

In sum, our data argue for the idea that antibody variants with an ability to bind TNFR2 on two neighboring cells, such as constructs with oppositely oriented TNFR2 binding sites or constructs with unidirectional oriented but highly movable binding sites, robustly stimulate full TNFR2 signaling while variants with six or more unidirectionally oriented, spatially less flexible binding sites preferentially engage the classical NFκB pathway and less the alternative NFκB pathway.

### Antibody based TNFR2 agonists with FcγR-independent activity expand regulatory T-cells ex vivo and in vivo

**Figure 6:**
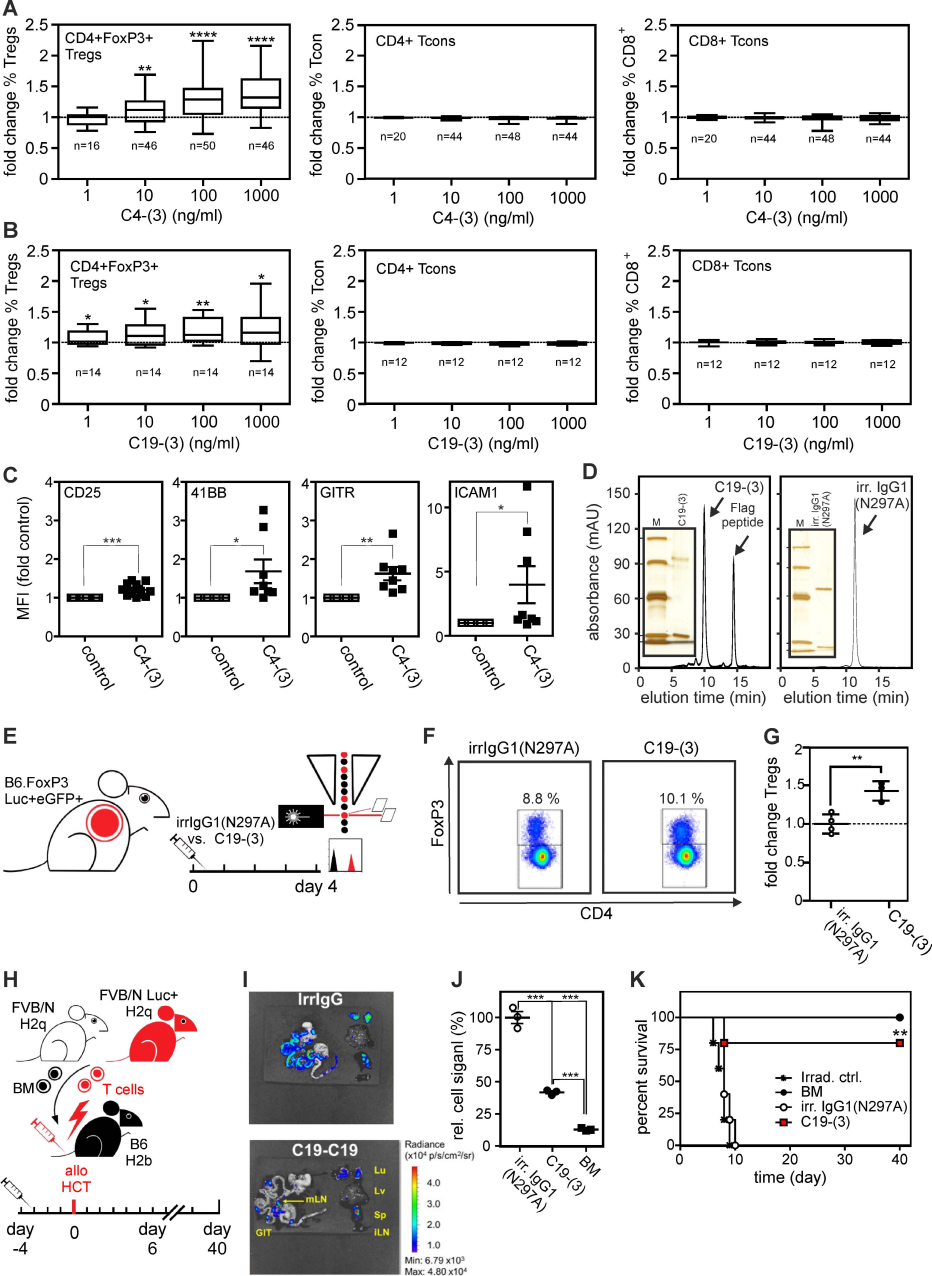
** Format (3) agonists induce Treg expansion in vitro, in vivo and and protect from acute GvHD.** (**A,B**) Change of frequencies of CD3^+^CD4^+^FoxP3^+^ Tregs, CD3^+^CD4^+^FoxP3^-^ Tcons and CD3^+^CD8^+^FoxP3^-^ Tcons in human peripheral blood mononuclear cells (PBMCs) after 4 days of stimulation with (**A**) anti-human TNFR2 C4-(3) and (**B**) anti-human/mouse TNFR2 cross-reactive C19-(3) agonists relative to untreated control samples of the same donor. Number of individual donors analyzed are indicated with n; * p < 0.05, **p < 0.01, **** p < 0.0001. (**C**) Fold change of expression of Treg activation markers measured with flow cytometry relative to the corresponding untreated controls of the same donor. * p < 0.05, **p < 0.01, **** p < 0.0001. (**D**) Purified C19-(3) and the irrelevant control antibody irrIgG1(N297A) were analyzed by gel filtration. Inserts show the purified proteins and marker proteins (M) separated by SDS-PAGE and visualized by silver staining. Marker positions correspond to 97, 66, 45, 30 and 20.1 kDa. (**E-G**) C19-(3) increases mouse Treg pool in vivo. (**E**) Experimental set-up: FoxP3.Luci.eGFP-DTR reporter mice were treated i.p. with C19-(3) or an irrelevant human IgG1 antibody (irrIgG1(N297A) and after four days splenic Treg frequencies were measured by flow cytometry. (**F**) Representative histogramms. (**G**) Averaged flow cytometric results. The individual Treg frequencies measured in % were normalized against the average of the three independent irrIgG1(N297A) control samples. ** *p*-value < 0,01. (**H-K**) C19-(3) protects from GvHD. (**H**) Experimental set-up: B6.WT mice were injected with C19-(3) or an irrelevant human IgG1(N297A) antibody 4 days before allo-HCT (9 Gy myeloablative conditioning, 5×10^6^ allogeneic FVB/N bone marrow cells (BM) plus luciferase^+^ 1,2×10^6^ FVB.Luc^+^ T-cells). (**I**) Representative ex vivo bioluminescence images of GvHD target organs, spleen and inguinal lymph nodes on day 6 after allo-HCT reveal suppression of allreactive donor T cell expansion and target organ infiltration. (**J**) Normalized relative radiance of transplanted donor T-cells calculated as the change over the mean radiance (p/s/cm^2^/sr) in untreated (irrelevant IgG1-treated) recipient mice in the gastrointestinal tract subtracted by the background signal without organs and subtracted by the signal in mLN. n=3 per group. (**K**) Kaplan-Meier survival graph. Log-rank test, * p-value < 0,05.

Next, we evaluated the ability of C4-(3) and C19-(3) to increase Treg frequency in 4-day human high-density PBMC cultures. C4-(3) increased Treg frequency with a half maximal dose in the range of 10 ng/ml (**Figure 6A**). In human PBMCs 1,000 ng/ml C4-(3) expanded Tregs ~1.4 fold, similar to ligand-based agonists in vitro 15,16. C19-(3) appeared a bit less active but still significantly increased Treg frequency starting already at concentrations around 1 ng/ml (**Figure 6B**). There was no significant effect of the two type (3) constructs on the frequency of conventional CD4^+^ and CD8^+^ T-cells (**Figure 6A,B**). TNFR2 agonist-treatment concomitantly increased expression of Treg activation markers, such as CD25, 4-1BB, GITR and ICAM-1 (**Figure 6C**).

Last but not least, we exploited the human/mouse cross-reactivity of antibody C19 to evaluate the ability of construct type (3) to induce Treg expansion in vivo. Previously, we have shown that the TNFR2-selective murine TNF-based TNFR2 agonist STAR2 expands Tregs in mice and protects from allo-HCT-induced acute GvHD in a Treg-dependent manner 16. Therefore, we initially tested whether 19-(3) would expand Tregs in FoxP3.Luci-DTR reporter mice in vivo and injected 250 µg of purified C19-(3) or an irrelevant human IgG1(N297A) intraperitoneally (**Figure 6D,E**). Four days later, we analyzed splenocytes with flow cytometry to determine Treg frequency, which moderately but significantly increased in the TNFR2 agonist treated mice (**Figure 6F,G**). To examine whether the moderate C19-(3)-induced Treg expansion is sufficient to elicit therapeutic activity, we injected B6.WT mice with C19-(3) or an irrelevant human IgG1(N297A) antibody 4 days before allo-HCT (**Figure 6H**). C19-(3) treatment significantly reduced expansion of alloreactive T-cells in recipients transplanted with FVB/N bone marrow and luciferase^+^ donor T cells. Almost all mice treated with the C19-(3) were protected, while all controls treated with the irrelevant construct developed lethal acute GvHD (**Figure 6I-K**). The finding that the moderate C19-(3)-induced expansion of Tregs correlates with therapeutic activity is in good accordance with several previous studies with ligand-based TNFR2-specific agonists also reporting low Treg expansion but robust therapeutic activity in various disease models (for review see ref. 3). Conclusively, anti-TNFR2 variants with geometries showing in vitro intrinsic FcγR-independent agonism activate and expand Tregs and protect from GvHD in vivo.

## Discussion

TNFR2 clustering and signaling is naturally triggered by memTNF trimers and the mode of action of the latter can be mimicked by trimeric soluble TNF fusion proteins bound to a plasma membrane-localized target 11,23. The high local concentrations of ligand-bound TNFR2 trimers reached in the cell-to-cell contact zone suffice to promote spontaneous clustering via the weak intrinsic auto-affinity of TNFR2 24. A second method to enforce TNFR2 clustering is to physically link two or more TNF trimers, e.g. by genetic fusion with oligomerizing protein domains, to bring six or more TNFR2 molecules together in cis 13,15,25. We reasoned that bivalent anti-TNFR2 antibodies can act in a principally similar fashion. In line with this concept, we found here that anti-TNFR2 antibody variants with TNFR2 binding domains on opposing sides of a rigid molecular backbone (Fc, IgG1, TNC trimerization domain), which are prone to interact simultaneously with two TNFR2^+^ cells, and oligovalent anti-TNFR2 antibody variants with six or more TNFR2 binding domains regularly display strong FcγR-independent, thus molecule-intrinsic agonism (**Figures 1B,2B and Supplemental Figures S4,S5,S6**). For some of the constructs, especially those with scFv:TNFR2 domains, which have intramolecular high spatial freedom, agonism in trans as well as in cis appears possible. To experimentally verify finally to which extent certain construct types act agonistically in trans (simultaneous binding to two TNFR2^+^ cells) and/or in cis (binding only to TNFR2) more elaborated methods have measure TNFR2 activation at the single cell level clearly separating isolated cells. Notably the localization of the epitope recognized by an anti-TNFR2 antibody within the extracellular domain turned out to be largely irrelevant for the intrinsic agonism of a certain anti-TNFR2-antibody format (**Figure 3, Supplemental Figures S6,S7**). Our finding that the domain architecture of the TNFR2 binding sites of an anti-TNFR2 variant, rather than antibody-individual features, such as recognized epitope or affinity, is the decisive factor for FcγR-independent intrinsic agonism, tremendously impacts clinical development of antibody-based TNFR2 agonists in two ways. First, it delivers a rational basis to shift the focus during preclinical development from the classical evaluation of recognized epitope, isotype, affinity, etc. towards the identification of the best-suited domain architecture of an antibody-based TNFR2 agonist. Second, as the anti-TNFR2 antibody formats generated with the design principles summarized above display molecule intrinsic agonism, activities and limitations related to FcγR binding can be easily prevented by use of molecular scaffolds not interacting with FcγRs. Conventional anti-TNFR2 antibodies preclude this, as they promote no or only poor molecule intrinsic agonism and instead act as conditional dual agonists for TNFR2 and FcγRs! Of course, bona fide FcγR-independent authentic TNFR2 agonists, as developed in our study, are not useful when TNFR2 targeting is envisaged with the aim to trigger FcγR functions, e.g. deletion of Tregs by ADCC in tumor therapy.

Preclinical and clinical evaluation of bona fide TNFR2 agonists (ligand-based agonists or FcγR-independent agonistic anti-TNFR2 antibody variants) and FcγR-stimulating competent conventional anti-TNFR2 antibodies (**Supplemental Table S1**) must now show in which setting these two categories of TNFR2 targeting biologics elicit the most beneficial activity for patients. In case of the FcγR-independent agonistic anti-TNFR2 variants described in this study, it is self-understood that their further clinical development requires before some technical adoptions, such as removal of the Flag-tag and antibody humanization.

We were surprised by the finding that some anti-TNFR2 constructs displayed pathway-preferential/specific agonism. A subset of anti-TNFR2 constructs strongly induced IL8 production, which crucially requires classical NFκB pathway engagement, but elicited no, or only a poor, cytotoxic response in Kym-1 cells and inefficiently triggered p100 processing (**compare Figures 2B and 3 and Supplemental Figures S5,S6**). The other subset of constructs triggered all three downstream activities of TNFR2, and thus acted similar to membrane TNF and oligomerized TNF variants. Importantly, soluble TNF stimulates not any of the aforementioned TNFR2 activities 13,26. Recruitment of the TNFR2-interacting TRAF2 molecule and the TRAF2-interacting E3 ligases cIAP1 and cIAP2 is required for IL8 induction, but also for p100 processing and promotion of cell death, albeit with a different molecular mechanism. Recruitment of TRAF2 and cIAP1/2 and activation of the classical NFκB pathway by TNFR2 occurs rapidly and requires IKK complex activation (few minutes), while the two other events require hours of TNFR2 stimulation to become manifest and rely on limiting the available pool of TRAF2 and cIAP molecules for other processes 18. Therefore, it appears possible that there are pathway-specific thresholds for the strength and/or durability of TRAF2 recruitment needed to engage a distinct pathway. Since antibody-based agonists certainly form complexes with TNFR2, which differ in their dynamics and stability (T_1/2_), such pathway-specific thresholds could be differentially achieved dependent on the respective agonist construct type. The molecular basis for the pathway preferential agonism of some antibody formats remains to be clarified, but its potential relevance for improved or reduced efficacy in vivo in preclinical models and in the clinic warrants consideration in clinical development of TNFR2 agonists.

Notably, antibody-based TNFR2 agonists, in contrast to ligand-based TNFR2 agonists, do not interact with clinically approved TNF-neutralizing antibodies. This important difference allows to straightforwardly combine antibody-based TNFR2 agonists together with TNF-neutralizing antibodies, a promising therapeutic concept in cases where TNF blockers alone elicit no therapeutic efficacy. Indeed, TNF blockade fails or even exacerbates disease activity in TNF-driven pathologies, such as MS or heart failure, and significant patient numbers do not respond in approved applications where TNF blockers demonstrated high clinical efficacy 4,5. It is tempting to speculate that in these cases autonomous TNFR2 agonists could not only exert beneficial effects as monotherapies but may also act synergistically in combination treatment with TNF blockers. In such cases, exogenous TNFR2 stimulation with an antibody-based TNFR2 agonist, could render the various approved TNF-neutralizing antibodies into “phenotypically” specific TNFR1 inhibitors.

## Materials and Methods

**Mice.** C57BL/6 (“B6”) and FVB/N mice were from Janvier Labs (Le Genest Saint Isle, France). C57BL/6J-Tyrc-2J/Foxp3.Luci.DTR-4 (B6.FoxP3.Luci-DTR), and FVB/N.L2G85 (FVB.Luc^+^) mice were bred at the Center for Experimental Molecular Medicine (ZEMM) of the University of Würzburg, Germany. All mice were housed in a specific pathogen-free facility and experiments were carried out according to the German regulations and reviewed and approved by the governmental authorities (Regierung von Unterfranken, 55.2.2-2532-2-537-61).

**Cell lines and cell culture conditions.** HEK293T cells, HT1080-TNFR2 and Kym-1 cells were cultured in RPMI 1640 medium (Sigma-Aldrich, Steinheim, Germany) supplemented with 10 % heat-inactivated fetal bovine serum (FBS) (GIBCO, EU Approved, South America) at 5 % CO_2_, 37 °C.

**Molecular cloning, expression and purification of anti-TNFR2 variants.** DNA cassettes encoding the typically Flag-tagged light and heavy chain proteins listed in **Supplemental Table S2** were cloned in the expression vector pCR3 using standard cloning techniques, PCR and synthetic genes. To express the various anti-TNFR2 variants, HEK293 cells were transiently transfected with the plasmid combinations shown in **Supplemental Table S**3 using the PEI method as described elsewhere in detail 27. In brief, HEK293 cells were grown in 15 cm tissue culture dishes. When cells reached confluence, cell culture medium was replaced by 15 ml serum-free RPMI 1640 with 1 % penicillin-streptomycin (Sigma, Deisenhofen, Germany). In parallel, 12 µg of a 1:1 mixture of the expression plasmids encoding the light and heavy chain of the antibody of interest along with 36 µl of a 1 mg/ml polyethyleneimine (PEI, Polyscience Inc., Warrington, USA) stock solution were added to 2 ml serum-free RPMI 1640 medium, vortexed and incubated for 10 minutes at room temperature. The plasmid/PEI solution was then added to the HEK293 cells and the next day, the plasmid/PEI-containing medium was replaced by RPMI 1640 medium with 2 % FBS and 1% penicillin-streptomycin was added. After 4-6 days the cell culture supernatants were collected, cleared by centrifugation (10 min, 4630 g) and evaluated for antibody production western blotting with anti-hIgG1 or anti-Flag and a standard antibody of known concentration and Flag-tagged heavy and light chain.

For anti-Flag affinity purification of the anti-TNFR2 variants, anti-Flag mAb M2 agarose columns (column diameter, volume 1-4 ml; app. 1 ml M2 agarose per mg of Flag-tagged antibody) were packed by gravity flow and equilibrated twice with TBS (10 x column volume). Cell culture supernatants were supplemented with 1 % w/v NaCl and loaded on the columns prepared (app. drops per min). After washing the columns 3 times with 5-10 column volumes of TBS, Flag-tagged antibody fusion proteins were eluted with 8 aliquots of the column volume containing 100 µg/ml 3xFlag peptide (Sigma, Deisenhofen, Germany) in TBS. The purity of the eluted proteins was determined by SDS-PAGE and silver staining of the gel (Thermo Scientific, Rockford, USA). The concentration of the purified proteins was furthermore estimated by comparison with the proteins of known size and concentration of the “Low Molecular Weight Calibration Kit for SDS Electrophoresis” (GE Healthcare UK Limited, Little Chalfont, UK) which were coapplied to SDA-PAGE gel. The possible LPS contamination of purified proteins was estimated with the Pierce LAL Chromogenic Endotoxin Quantitation Kit (Thermo Fisher Scientific) as recommended by the manufacturer's protocol. If present, LPS was removed using the Pierce High Capacity Endotoxin Removal Resin as mentioned by the manufacturer.

**Size Exclusion Chromatography.** A MAbPac™ SEC-1 HPLC column (Thermo Scientific, Rockford, USA) was pre-equilibrated with PBS for 25 minutes at a flow rate of 0.76 ml/min. After the column pressure stayed stable, protein samples (100-200 µl, 100-500 µg/ml) were injected. Protein was detected using an UV detector at 280 nm. Calibration of the column was carried out with the column performance check standard aqueous SEC 1 solution (Phenomenex, Torrance, USA) containing bovine thyroglobulin (670 kDa), IgA (300 kDa), IgG (150 kDa), ovalbumin (44 kDa), and myoglobin (17 kDa).

**In vitro evaluation TNFR2 activation.** Expression of IL8 is dominantly regulated via the classical NFκB pathway. Inducible IL8 production can therefore be used as a simple and reliable measurable indicator of classical NFκB pathway activity. To determine the ability of the various anti-TNFR2 variants to trigger TNFR2-mediated IL8 production, HT1080-TNFR2 cells (20.000 cells per well of a 96-well plate) were grown overnight and stimulated the next day by exchange of the culture medium with culture medium supplemented with the antibodies of interest. The highly active TNF-based TNFR2-specific agonist TNC-sc(mu)TNF80 (STAR2) 16 served as a positive control and benchmark. Supernatants were finally analyzed for their IL8 content using the BD OptEIA™ IL8 ELISA kit from Biosciences (Heidelberg, Germany) according to the manufacturer's protocol. EC_50_ values were derived manually from the dose-response blots using a helping line indicating 50 % of the maximal induction achieved with the TNC-sc(mu)TNF80 benchmark. To evaluate the ability of the anti-TNFR2 antibody variants to trigger TNFR2-mediated sensitization for TNFR1-induced cell death, Kym-1 cells were cultivated overnight (20.000 cells per well of a 96-well plate) and were challenged the next day for additional 18 hours with the constructs of interest. TNC-sc(mu)TNF80 served again as a benchmark. Finally, viable cells were quantified by crystal violet staining. To evaluate p100 to p52 processing as a hallmark of alternative NFκB pathway activity, 5 x 10^5^ Kym-1 were seeded per well of a 12-well plate. Next day, cells were stimulated with the anti-TNFR2 antibody variants and after an additional day, cells were washed with PBS and harvested by scraping with a rubber policeman. After centrifugation (2 min, 14000 g) the cell pellet lysed in 4x Laemmli sample buffer, sonicated for 25 sec with maximal amplitude (UP100H Ultrasonic Processor, Hielscher, Germany) heated for 5 min at 95 °C, and cleared from remaining insoluble debris by centrifugation (2 min, 14000 g) to pellet. Cell lysates were separated by SDS-PAGE and after transfer to nitrocellulose p100/p52 were detected by western blotting (p100/p52 (#4882S, Cell Signaling), ß-actin (#22180326, Sigma), HRP-labeled anti-rabbit (#7074, Sigma), HRP-labeled rabbit anti-mouse (#P0260, Dako)).

**Binding studies.** For cellular equilibrium binding studies with human and murine TNFR2, HEK293 cells were transiently transfected with expression plasmids encoding human and mouse TNFR2 encoding or empty vector (EV) using the PEI method as described in the antibody production paragraph. Aliquots of TNFR2-expressing and EV transfectants were pairwise incubated with increasing concentration of GpL (*Gaussia princeps* luciferase) fusion proteins of the anti-TNFR2 antibody variant of interest. After 1 h incubation under standard cell culture conditions cells were washed 5 times with ice-cold PBS to remove unbound GpL fusion proteins. Cells were resuspended in 50 µl RPMI 1640 media supplemented with 0.5 % FCS, transferred into a 96-black well plate and cell-associated luciferase activity was detected with a LUMO luminometer (anthos Mikrosysteme GmbH, Friesoythe, Germany) directly after adding of coelenterazine (Carl Roth, Karlsruhe, Germany) to reach a concentration of 1.5 µM. Specific binding values were calculated by subtracting the unspecific binding (EV-transfected cells) from the total binding (human or murine TNFR2-transfected cells) values. The calculated values were fitted by non-linear regression using GraphPad Prism 5.

**In vitro expansion and characterization of human Tregs.** Human PBMCs were isolated from whole blood by density gradient centrifugation using Ficoll. 1x10^7^ PBMCs/ml were precultured for 2 days as described elsewhere 28. 1x10^5^ cells/well were then seeded in a 96-well plate and stimulated for 4 days with the indicated concentrations of TNFR2 agonists. Finally, cells were washed and processed for flow cytometry analysis. After staining with surface antibodies FoxP3 was stained intracellular using the FOXP3 Fixation/Permeabilization Buffer from Biolegend according to the protocol of the manufacturer.

**In vivo Treg expansion.** B6.FoxP3.Luci-DTR reporter mice were injected intraperitoneally with 250 μg of the anti-TNFR2 variant C19-(3) or an isotype control antibody in PBS. Four days later, single cell suspensions were obtained from each spleen and then Treg frequencies were determined by flow cytometry in an AttuneNxT (Invitrogen) flow cytometer and later analyzed with the FlowJo software package (Tree Star). Staining of extracellular markers and FoxP3 was performed as described for the in vitro expansion assays.

**Acute GvHD (major mismatch) model.** 8-12 weeks old B6 (H-2K^b^) wild type mice were myeloablatively irradiated with 9 Gy in a Faxitron CP-160 X-ray machine. On the same day, bone marrow cells (BM) from FVB/N (H-2K^q^) mice and total T-cells from FVB.Luc^+^ mice, isolated with the Dynabeads Untouched Mouse T-cell kit (Invitrogen Ref. 11413D), were adjusted at a concentration of 50 and 6 million cells per milliliter. Within 4 hours, 5x10^6^ BM cells and 6x10^5^ T-cells were injected intravenously into the irradiated mice. On day 6 after transplantation, some mice were treated with 300 mg/kg bodyweight of D-Luciferin (injected intraperitoneally) and sacrificed 10 min later. Selected organs of these animals were prepared and placed on a black imaging plate to acquire bioluminescence images with an IVIS Spectrum CCD-imaging system (Perkin-Elmer) for analysis using the Living image software. Exposure time for each picture was set to 5 minutes at medium binning settings. Relative T-cell signal for each organ was quantified as the change in relative radiance normalized by the signal from untreated mice (isotype control) minus background signal from the black imaging plate. Animals not dedicated for imaging were evaluated till 40 days after transplantation or sacrificed after reaching the critical GvHD score (human endpoint).

## Supplementary Material

Supplementary figures and tables.
