# Supplementary material for: Generic design principles for antibody-based tumour necrosis factor (TNF) receptor 2 (TNFR2) agonists with FcγR-independent agonism
Source: Theranostics. 2024 Jan 1;14(2):496–509. doi: 10.7150/thno.84404 (PMC10758050; doi:10.7150/thno.84404)
Supplement: Supplementary file 1 — Supplementary figures and tables. [file thnov14p0496s1.pdf]

SUPPLEMENTAL DATA

Identification of generic design principles for antibody based tumor necrosis factor (TNF) receptor 2 (TNFR2) agonists with FcγR-independent agonism

Mohamed A. Anany[1,2], Stefanie Haack[3], Isabell Lang[1], Julia Dahlhoff[3], Juan Gamboa Vargas[3], Tim Steinfatt[3], Lea Päckert[1], Daniela Weisenberger[1], Olena Zaitseva[1], Juliane Medler[1], Kirstin Kucka[1], Tengyu Zhang[1], Tom Van Belle[4], Luc van Rompaey[4], Andreas Beilhack[3,*] and Harald Wajant[1,*]

[1] Division of Molecular Internal Medicine, Department of Internal Medicine II, University Hospital Würzburg, Würzburg, Germany.
[2] Department of Microbial Biotechnology, Institute of Biotechnology, National Research Center, Dokki, Giza, Egypt.
[3] Department of Internal Medicine II, University Hospital Würzburg, Würzburg, Germany.
[4] Dualyx NV, 9052 Zwijnaarde, Belgium
* equal contribution

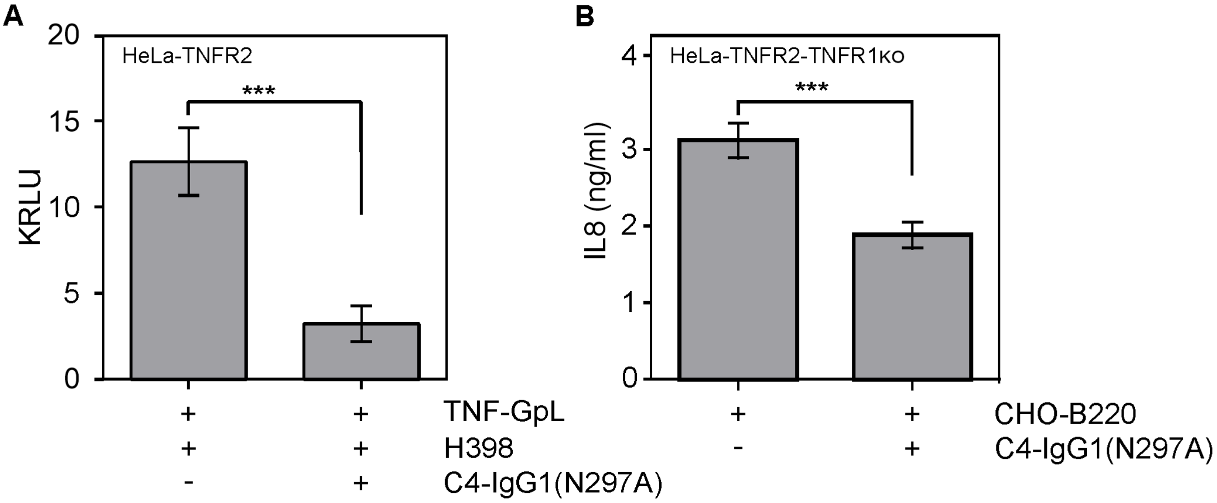

Supplemental Figure S1. **C4 is a TNFR2 antagonist.** (**A**) HeLa-TNFR2 cells were pretreated with 4 µg/ml of C4-IgG1(N297A) and were then incubated with 20 ng/ml of GpL-TNF at 37°C for 1 h. After removal of unbound GpL-TNF molecules, cell associated GpL-TNF was quantified by analysis of the cell attached luciferase activity. To prevent TNFR1 binding, cells were analyzed in the presence of 10 µg/ml of the TNFR1-blocking antibody H398. (**B**) HeLa-TNFR2-TNFR1$_{KO}$ cells were stimulated with membrane TNF-expressing CHO cells (CHO-B220) in the presence of 4 µg/ml of C4-IgG1(N297A) and next day TNFR2-mediated IL8 production was quantified by ELISA. Shown are the results of ten independent experiments in (A) and of six independent experiments in (B). ***, $p < 0.001$; repeated measures ANOVA.

A

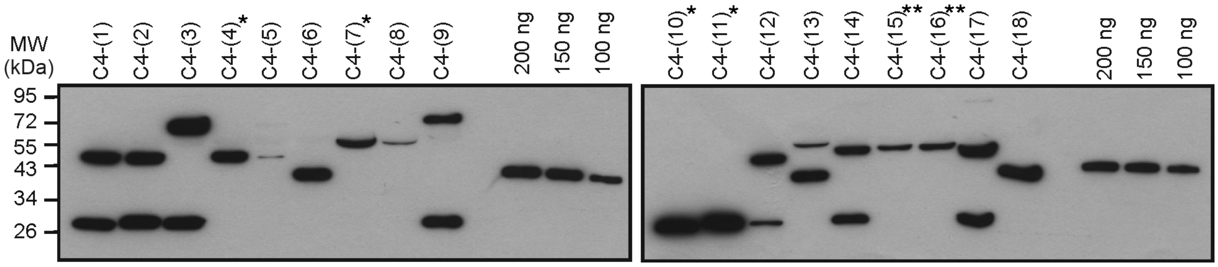

* constructs with two chains of similar size

** constructs composed of only one type of chain

B

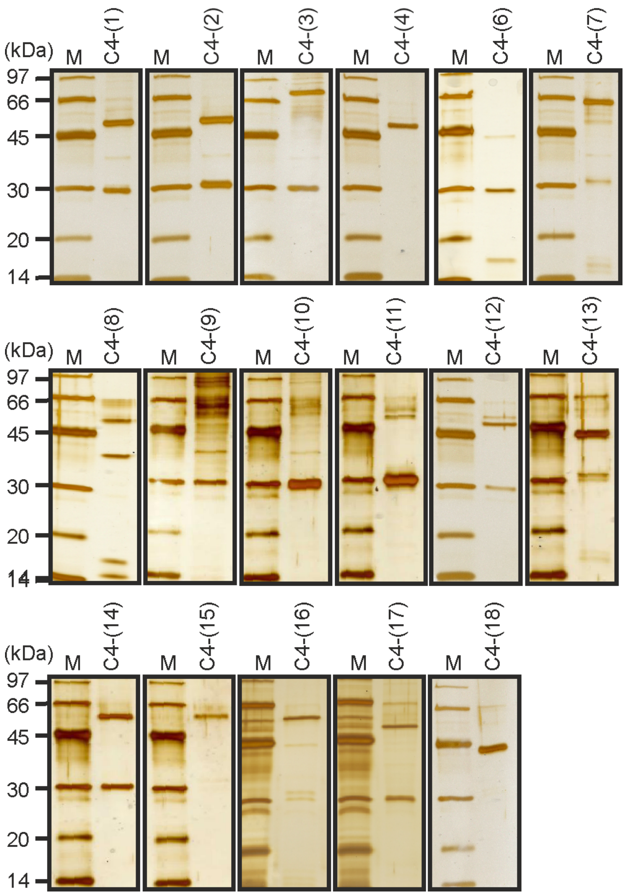

C

**Construct**	**Concentration (ng/ml)**	
	Individual experiments	Average
C4-(1)	50 / 50 / 40	47
C4-(2)	50 / 50 / 40	47
C4-(3)	60 / 60 / 60	60
C4-(4)	20 / 5 / 10	12
C4-(5)	~2 / < 2 / <2	< 2
C4-(6)	20 / 10 / 20	17
C4-(7)	15 / ~ 2 / 20	12
C4-(8)	~2 / 5 / 5	4
C4-(9)	40 / 50 / 40	43
C4-(10)	40 / 30 / 30	37
C4-(11)	40 / 30 / 30	33
C4-(12)	30 / 15 / 30	25
C4-(13)	30 / 20 / 30	27
C4-(14)	40 / 40 / 40	40
C4-(15)	10 / 10 / 10	10
C4-(16)	10 / 10 / 10	10
C4-(17)	50 / 30 / 40	40
C4-(18)	30 / 20 /20	23

Supplemental Figure S2. **Transient expression of C4-derived variants C4-(1) to C4-(18).** (**A**) Cell culture supernatants of HEK293 cells transiently producing the indicated C4 antibody variants along with the indicated amounts of a Flag-tagged standard protein were analyzed by western blotting using the anti-Flag mAb M2 and HRP-labeled rabbit anti-mouse (#P0260, Dako). One of three independent production campaigns is shown. (**B**) Flag-tagged proteins contained in the supernatants from A were affinity purified on Flag agarose and analyzed by SDS-PAGE. Proteins in the gel were visualized by silver staining. Due to its low productivity C4-(5) was not purified. (**C**) Productivity of transiently produced C4 variants. Supernatants were subjected to western blotting as in A and the concentrations of the various C4 variants were estimated by comparison with the Flag-tagged standard.

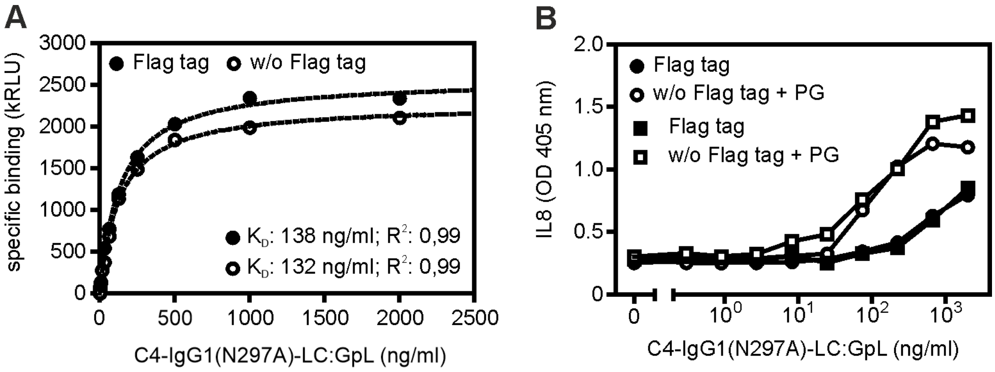

Supplemental Figure S3. **N-terminal Flag tagging of C4 does not affect its interaction with TNFR2.** (**A**) HT1080 and TNFR2-expressing HT1080 transfectants were seeded in a black 96 well tissue culture plate. The next day, wells containing the 2 cell types were pairwise incubated (1 h, 37°C) with increasing concentrations of C4-IgG1(N297A)-LC:GpL with and without N-terminal Flag tags. After removal of unbound protein molecules, cell-associated luciferase activity was measured as described in the bindung studies method paragraph. Specific binding values for the two C4-(IgG1(N297A)-LC:GpL variants were calculated by subtracting the non-specific binding values derived of the HT1080 cells from the total binding values derived from the TNFR2 transfectants. K_D values were obtained using the "nonlinear regression to a one-site specific binding curve" function of the GraphPad Prism5 software. Shown is one representative experiment of 3. (**B**) HT1080 cells and HT1080-TNFR2 transfectants were stimulated in triplicates with increasing concentrations of the indicated C4 variant in the presence and absence of 1 µg/ml protein G (PG) as „crosslinker". Next day, supernatants were investigated for the presence of IL8 by ELISA.

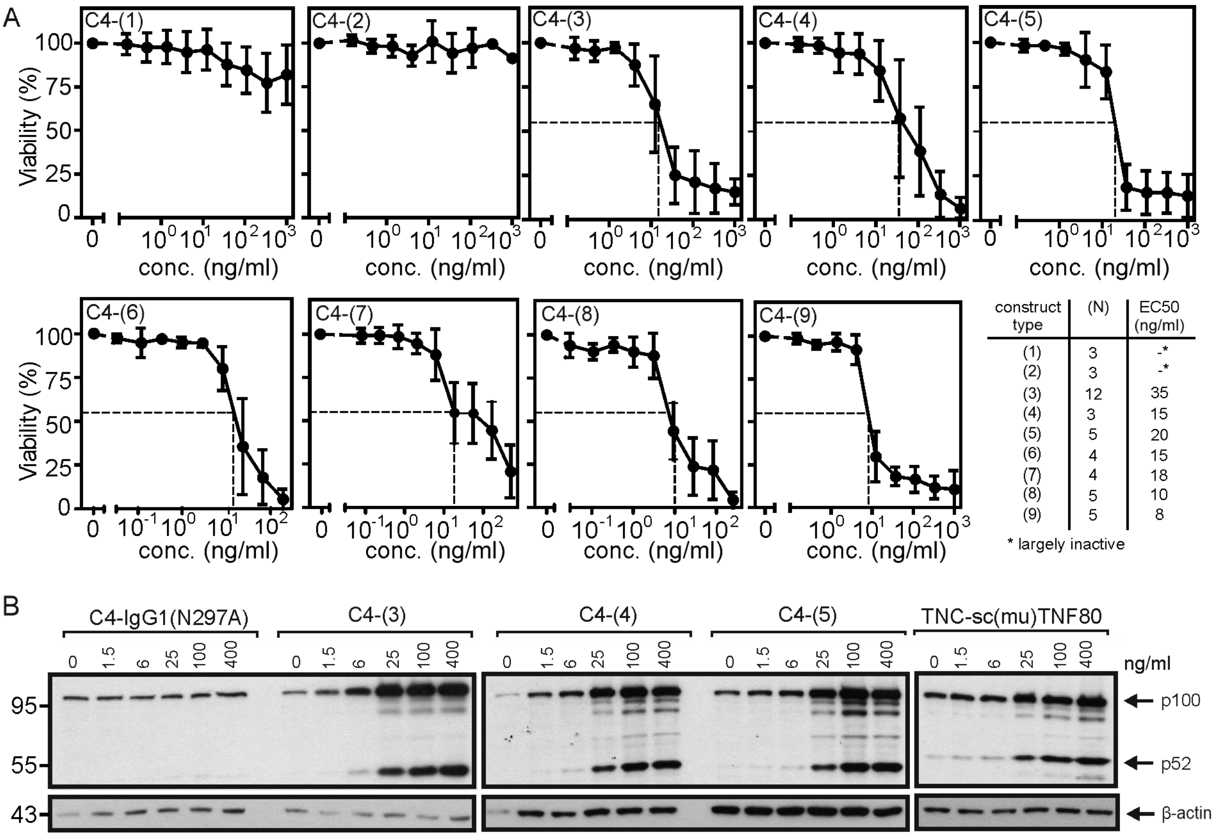

Supplemental Figure S4. **Ability of C4 antibody variants with N- and C-terminal TNFR2 binding sites to induce Kym-1 cell death and to trigger p100 processing.** (**A**) Kym-1 cells were treated with the various C4 constructs and the next day cell viability was determined by crystal violet staining. Maximal cell death induction by TNC-sc(mu)TNF80 is indicated by a dotted line. Shown are averaged data of 3–12 independent experiments. The number of experiments for each construct and their EC50 values are listed in the table. (**B**) Kym-1 cells were again stimulated overnight with the indicated C4 variants. To prevent cell death induction 20 µM ZVAD was added. Total cell lysates were analyzed by western blotting with respect to p100 processing .

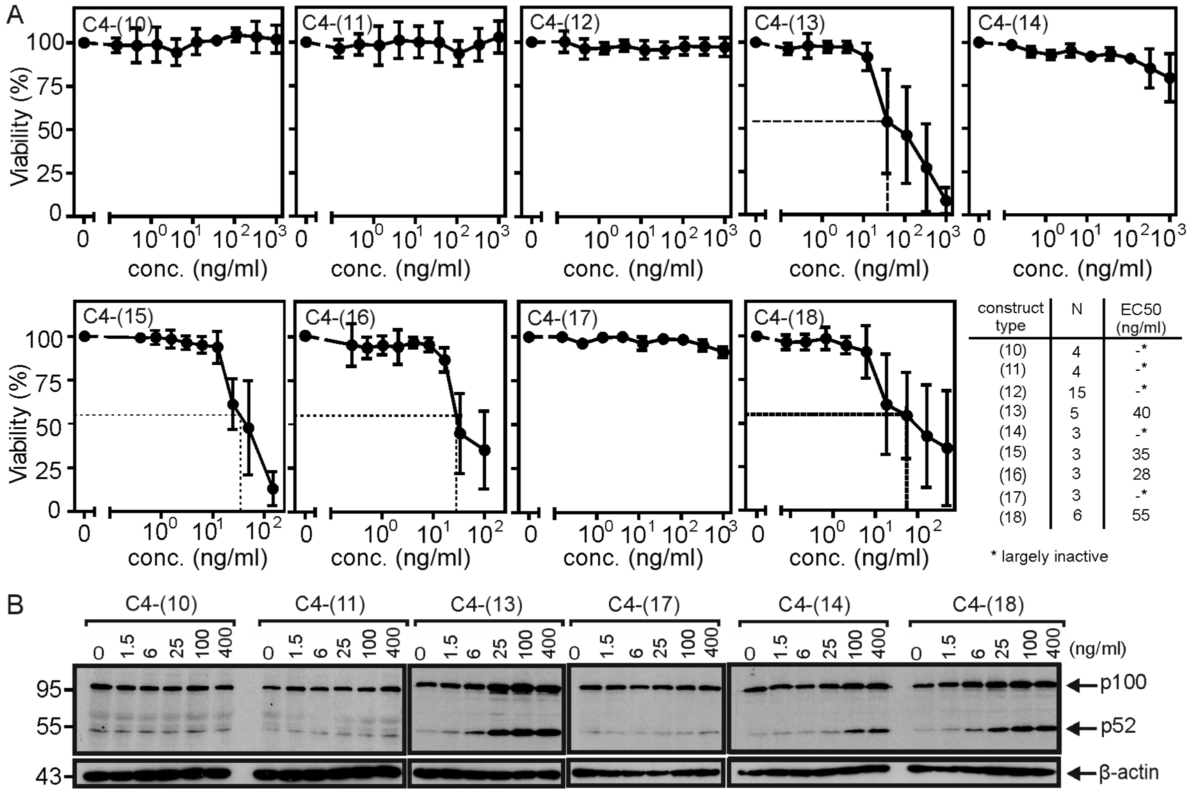

construct type	N	EC50 (ng/ml)
(10)	4	-*
(11)	4	-*
(12)	15	-*
(13)	5	40
(14)	3	-*
(15)	3	35
(16)	3	28
(17)	3	-*
(18)	6	55

Supplemental Figure S5. **Ability of C4 antibody variants with unidirectional oriented N-terminal TNFR2 binding sites to induce Kym-1 cell death and to trigger p100 processing.** (**A**) Kym-1 cells were challenged with the C4 constructs and cell viability was determined the next day by crystal violet staining. The dotted lines show the EC50 of each antibody depending on the maximal cell death induction by TNC-sc(mu)TNF80. (**B**) Total lysates of Kym-1 cells stimulated overnight with the indicated C4 constructs were analyzed by western blotting.

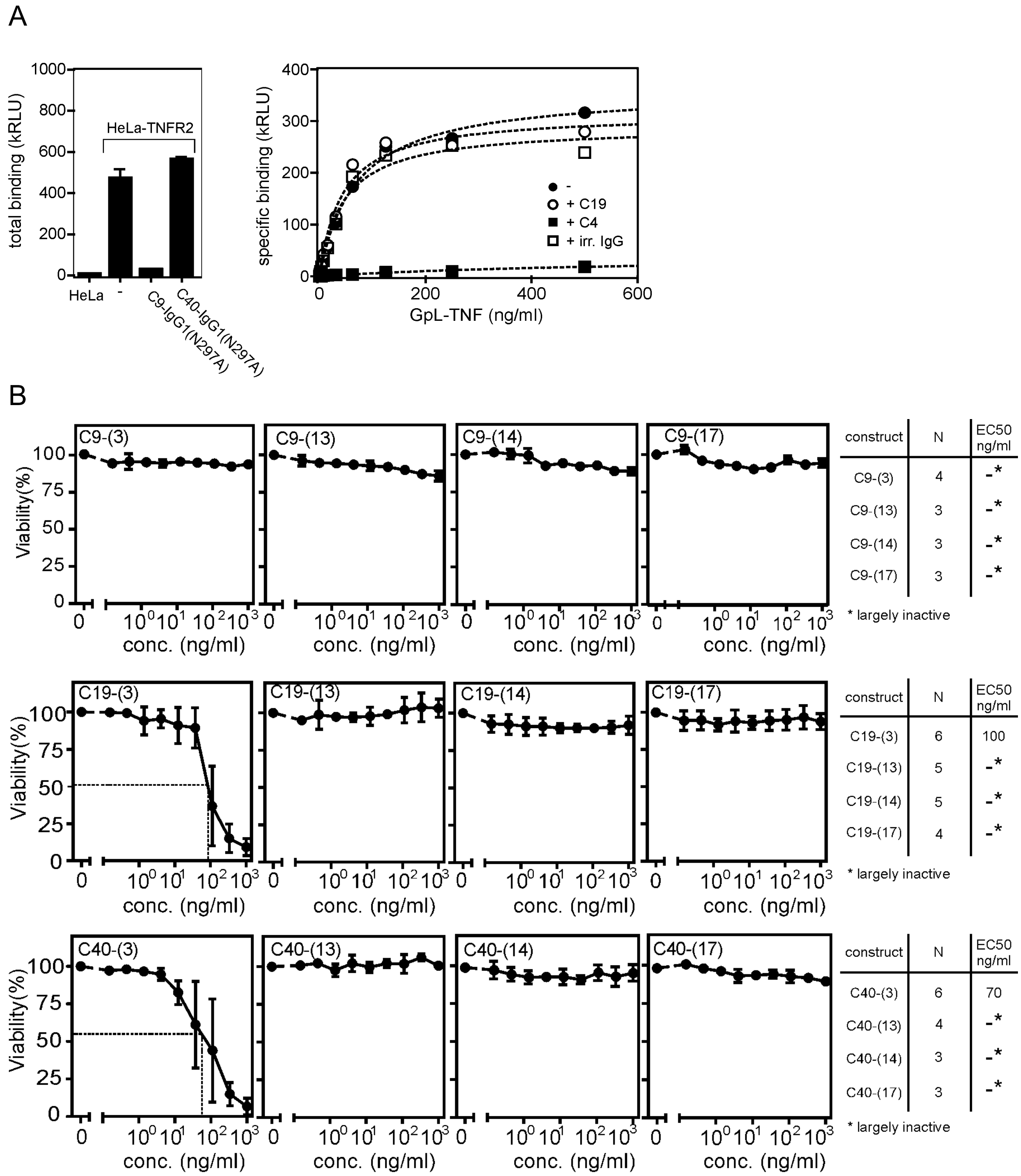

construct	N	EC50 ng/ml
C9-(3)	4	-*
C9-(13)	3	-*
C9-(14)	3	-*
C9-(17)	3	-*

* largely inactive

construct	N	EC50 ng/ml
C19-(3)	6	100
C19-(13)	5	-*
C19-(14)	5	-*
C19-(17)	4	-*

* largely inactive

construct	N	EC50 ng/ml
C40-(3)	6	70
C40-(13)	4	-*
C40-(14)	3	-*
C40-(17)	3	-*

* largely inactive

Supplemental Figure S6. **Intrinsic agonism of anti-TNFR2-antibody variants of format (3), (13), (14) and (17) to induce cell death**. (**A**) Effect of anti-TNFR2 antibodies C9, C19 and C40 on ligand binding. Left panel: HeLa-TNFR2 cells were pretreated with C9-IgG1(N297A) (5 µg/ml) or C40-IgG1(N297A) (3 µg/ml) and were then incubated with 5 ng/ml of GpL-TNF at 37°C for 1 h. After removal of unbound GpL-TNF molecules, cell associated GpL-TNF was quantified by analysis of the cell attached luciferase activity. Parental HeLa cells, not expressing TNFR2, served as control for non-specific binding. Right panel: HT1080-TNFR2 cells were seeded in a black 96 well tissue culture plate. The next day, cells were preincubated with 5 µg/ml of C4-IgG1(N297A), C19-IgG1(N297A) or an irrelevant human IgG1. Cells were incubated for 1 h at 37°C) with increasing concentrations of GpL-TNF and after removal of unbound protein molecules, cell-associated luciferase activity was measured. (**B**) Kym-1 cells were stimulated with the indicated anti-TNFR2 variants and the next day cell viability was determined by crystal violet staining.

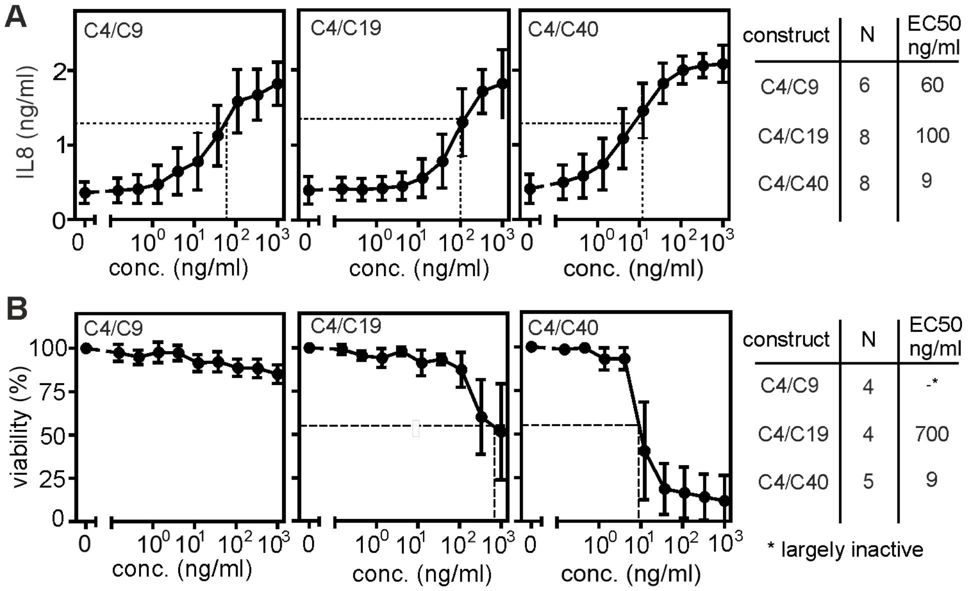

Supplemental Figure S7. **Intrinsic agonism of biparatopic anti-TNFR2 variants of format (3) and (13).** (**A**) HT1080-TNFR2 cells were stimulated with the indicated anti-TNFR2 antibody variants overnight and IL8 production was quantified by ELISA. The half maximal TNFR2 response level induced by TNC-sc(mu)TNF80 is indicated by a dotted line. Averaged data of 6 – 8 independent experiments are shown. (**B**) Kym-1 cells were stimulated with the indicated anti-TNFR2 variants and analyzed for cell death induction by crystal violet staining.

A

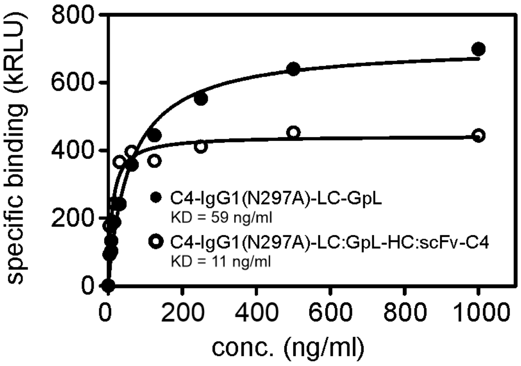

B

Exp. no	affinity (ng/ml) C4-	C4-...-C4
1	59	11
2	49	2
3	45	24
4	13	9
5	70	22
average	47	14

Supplemental Figure S8. **Apparent affinity of GpL variants of C4-IgG1(N297A) and C4-IgG1(N297A)-HC:scFvC4.** HeLa cells and HeLa-TNFR2 cells were cultivated overnight in 24-well plates (half-plate Hela cells and half-plate HeLa-TNFR2 cells transfectants). The two HeLa variants were pairwise incubated with the indicated concentrations of C4-IgG1(N297A)-LC:GpL and C4-(3)-LC:GpL for one hour at 37 °C. After removal of unbound proteins, nonspecific (HeLa) and total (HeLa-TNFR2) cell-associated binding of the two GpL fusion proteins was quantified by measuring GpL activity. Unspecific binding values were subtracted from the corresponding total binding values to calculate specific binding values which were fitted by non-linear regression to a single binding site interaction plot with the GraphPad Prism5 software. One representative experiment is shown in (A). Affinities from 5 independent experiments were listed in the table shown in (B).

Supplemental Table S1: Biochemical and functional properties of published anti-TNFR2 antibodies

Antibody / isotype	Effect on TNF binding	FcγR-independent activation/inhibition of TNFR2*	Effect of crosslinking or FcγR-binding		Ref. ****
			In vitro	In vivo	
Ty101 anti-mTNFR2 ratIgG1	Blocking	Inhibits TNF-induced Treg proliferation	Not verified	Not verified	1)
UTR1 anti-hTNFR2 mIgG1	Blocking	Inhibits TNF-induced GM-CSF production	Agonistic after crosslinking	Not verified	2,3)
TR75-54.7 anti-mTNFR2 hamster IgG	Blocking	Inhibits TNF-induced CT6 proliferation	Agonistic after crosslinking binds mFcγRII and FcγRIII	Inhibits CT26 tumors	4,5)
An3025 anti-hTNFR2 rabbit mAbY hIgG1	Blocking	Inhibits TNF-induced TNFR2 activation	ADCC	Inhibits MC38 in hTNFR2ki-mice ADCC required	6) WO2022/122005A1 Adlai Northy
Y9 anti-mTNFR2 mIgG2a	Blocking despite binding CRD1	Not TNFR2 stimulating	Agonistic when plate-bound ADCC	Inhibit tumor FcγR-binding required	7)
Ab1 and Ab2 ABV2c anti-hTNFR2 hIgG1	Blocking	Not investigated	T-cell costimulation when plate-bound, dito NFκB reporter	Not verified	7) WO2020/061210A1 Merrimack
M861 mIgG1	Presumably Blocking	Inhibits TNF-induced proliferation of IL2-treated T-cells	Not verified	Inhibits CT26 tumors in combination with CpG ODN, FcγR relevance not checked	8)
MR2-1 anti-hTNFR2 mIgG1	-	Weak IL8 induction	Potentiated IL8 induction in presence of FcγIIb	Not verified	9)
F10, A05 anti-mTNFR2<br><br>B02, …, H10 A09, …, H03 anti-hTNFR2 Hu/mu IgG2a and IgG1	Blocking (e.g. F10) Non-blocking (e.g. A05)	Non-blocking enhance and blocking antibodies inhibit NK cytokine release, problem synergism with endogenous TNF	Not verified	FcγR-dependent anti-tumor activity	WO2020/089473A2 WO2020/089474A1 Bioinvent
BI-1808 anti-hTNFR2	Blocking	Not investigated	Not verified	FcγR-dependent Treg depletion	10)
HFB200301 anti-hTNFR2 hIgG1	Non-blocking	Low activation of CD8 and NK cells but strongly enhanced by sTNF	Not verified	Anti-tumor activity	11) WO2021/141907A1 HifiBio
SIM0235 anti-hTNFR2	Blocking	Not investigated	ADCC, ADCP	Not verified	WO2021/023098A1**

hIgG1					Simcere
SBT-001 – SBT-004 anti-hTNFR2 various IgGs	Blocking	Inhibits TNF-induced Treg proliferation	Not verified	Not verified	US10988543 B2 OPI-VI
TNFRAB1, TNFRAB2	-	Inhibits TNF-induced Treg proliferation	No effect on TNFR2 inhibition	Not verified	12) AU2017/263833A1 General Hospital Corporation
E4 anti-hTNFR2 IgG1	Non-blocking	Neutral on TNF-induced Treg proliferation	ADCC-dependent Treg depletion***	Not verified	WO2018/213064A1 NIH
C4, C15, C16, C17, K21, C27, C40 covering (CRD1, CRD2, CRD3, CRD4) anti-hTNFR2 hIgG1, for C4 also hIgG2, hIgG3, hIgG4	Blocking (C4) and non-blocking (C40); this ms.	No activity per se, C4 inhibits ligand-induced IL8 (this ms.)	FcγR-dependent, IL8 induction	Not verified	9)
55F6, 25-71, 25-81 and many more anti-hTNFR2 hIgG1	> 30 blocking 10 non-blocking	Only 55F6 poor NFκB reporter (< 0.2 ng/ml TNF)	ADCC	Reduced growth of TNFR2^{high} Colo205 cells (xenograft model)	WO2021/055253A2 Apexigen
30.083, 30.091 anti-hTNFR2 hIgG1	Not tested	Neutral on TNF-induced NFκB reporter	Not verified	Not tested	WO2022/003693A1 Biolojic
R2-1…R2-6, anti-hTNFR2 hIgG1	R2-1 weakly all other Abs strongly Blocking	Blocking of TNF-induced NFκB reporter	NFκB reporter activation in presence of FcγR cells	Inhibits MC38 tumors in TNFR2ki (mIgG1, mIgG2a) reduced activity with mIgG1D265A	WO2022/147222A1 Novarock
Hu32-C, hu3-E, anti-hTNFR2	Blocking	Not verified	T-cell costimulation when plate-bound ADCC	No verified	WO2021/249542A1 Nanjing leads biolabs

* Use of TNFR2 responding cells w/o FcγR expression or use of FcγR-defective IgG variants
** Patent describes several antibodies with general similar activities, concrete identity of SIM0235 is not evident
*** Demonstrated by use of defucosylated IgG1 (FUT8-deficient CHO produced)
**** S1 table references:

1) Jiang M, Liu J, Yang D, Tross D, Li P, Chen F, Alam MM, Faustman DL, Oppenheim JJ, Chen X. A TNFR2 antibody by countering immunosuppression cooperates with HMGN1 and R848 immune stimulants to inhibit murine colon cancer. Int Immunopharmacol. 2021 Dec;101(Pt A):108345.

2) Brockhaus M, Schoenfeld HJ, Schlaeger EJ, Hunziker W, Lesslauer W, Loetscher H. Identification of two types of tumor necrosis factor receptors on human cell lines by monoclonal antibodies. Proc Natl Acad Sci U S A. 1990 Apr;87(8):3127-31.
3) Vandenabeele P, Declercq W, Vercammen D, Van de Craen M, Grooten J, Loetscher H, Brockhaus M, Lesslauer W, Fiers W. Functional characterization of the human tumor necrosis factor receptor p75 in a transfected rat/mouse T cell hybridoma. J Exp Med. 1992 Oct 1;176(4):1015-24.
4) Williams GS, Mistry B, Guillard S, Ulrichsen JC, Sandercock AM, Wang J, González-Muñoz A, Parmentier J, Black C, Soden J, Freeth J, Jovanović J, Leyland R, Al-Lamki RS, Leishman AJ, Rust SJ, Stewart R, Jermutus L, Bradley JR, Bedian V, Valge-Archer V, Minter R, Wilkinson RW. Phenotypic screening reveals TNFR2 as a promising target for cancer immunotherapy. Oncotarget. 2016 Oct 18;7(42):68278-68291.
5) Sheehan KC, Pinckard JK, Arthur CD, Dehner LP, Goeddel DV, Schreiber RD. Monoclonal antibodies specific for murine p55 and p75 tumor necrosis factor receptors: identification of a novel in vivo role for p75. J Exp Med. 1995 Feb 1;181(2):607-17.
6) Chen Y, Jia M, Wang S, Xu S, He N. Antagonistic Antibody Targeting TNFR2 Inhibits Regulatory T Cell Function to Promote Anti-Tumor Activity. Front Immunol. 2022 Feb 16;13:835690.
7) Tam EM, Fulton RB, Sampson JF, Muda M, Camblin A, Richards J, Koshkaryev A, Tang J, Kurella V, Jiao Y, Xu L, Zhang K, Kohli N, Luus L, Hutto E, Kumar S, Lulo J, Paragas V, Wong C, Suchy J, Grabow S, Dugast AS, Zhang H, Depis F, Feau S, Jakubowski A, Qiao W, Craig G, Razlog M, Qiu J, Zhou Y, Marks JD, Croft M, Drummond DC, Raue A. Antibody-mediated targeting of TNFR2 activates CD8+ T cells in mice and promotes antitumor immunity.Sci Transl Med. 2019 Oct 2;11(512):eaax0720.
8) Nie Y, He J, Shirota H, Trivett AL, Yang D, Klinman DM, Oppenheim JJ, Chen X. Blockade of TNFR2 signaling enhances the immunotherapeutic effect of CpG ODN in a mouse model of colon cancer.Sci Signal. 2018 Jan 2;11(511):eaan0790.
9) Medler J, Nelke J, Weisenberger D, Steinfatt T, Rothaug M, Berr S, Hünig T, Beilhack A, Wajant H. TNFRSF receptor-specific antibody fusion proteins with targeting controlled FcγR-independent agonistic activity.Cell Death Dis. 2019 Mar 4;10(3):224.
10) Linda Mårtensson, Kirsty Cleary, Petra Holmkvist, Mathilda Kovacek, Carolin Svensson, Monika Semmrich, Therese Blidberg, Mimoza Demiri, Osman Dadas, Marie Borggren, Vici Pitic, Sean H. Lim, Stephen A. Beers, Susanne Gertsson, Kristoffer S. Rohrberg, Ingrid Karlsson, Andres McAllister, Mark S. Cragg, Björn Frendeus, Ingrid Teige. BI-1808 - a first in class ligand-blocking αTNFR2 antibody for cancer immunotherapy [abstract]. In: Proceedings of the American Association for Cancer Research Annual Meeting 2022; 2022 Apr 8-13. Philadelphia (PA): AACR; Cancer Res 2022;82(12_Suppl):Abstract nr 4156.
11) Shuo Wei, Ross Fulton, Yun-Yueh Lu, Qian Zhang, He Zhou, Andreas Raue, Mingjie Chen, Wenhua Xu, Xing Cai, Juliana Crivello, Zachary Duda, Zhiyuan Wang, Rebecca Silver, Alexandra Staskus, Charina Ortega, Sami Ellouze, Carine George, Sophie Foulon, Dean Lee, Monika Manne, Nicola Beltraminelli, Jinping Gan, Francisco Adrian, Liang Schweizer, Jennifer Watkins-Yoon. Mechanism of action and biomarker strategy for HFB200301, an anti-TNFR2 agonist antibody for the treatment of cancer [abstract]. In: Proceedings of the American Association for Cancer Research Annual Meeting 2021; 2021 Apr 10-15 and May 17-21. Philadelphia (PA): AACR; Cancer Res 2021;81(13_Suppl):Abstract nr 1883.
12) Torrey H, Butterworth J, Mera T, Okubo Y, Wang L, Baum D, Defusco A, Plager S, Warden S, Huang D, Vanamee E, Foster R, Faustman DL. Targeting TNFR2 with antagonistic antibodies inhibits proliferation of ovarian cancer cells and tumor-associated Tregs.Sci Signal. 2017 Jan 17;10(462):eaaf8608.

Supplemental Table S2. AA sequence of light and heavy chain proteins used in the study. Leader, underlined; Linker sequences, bold; Flag tag, underlined + grey background; variable domains, italic; constant IgG1 domains, grey background; TNC trimerization domain, italic + underlined + grey background.

Plasmid No.	Encoded peptide	AA sequence (FASTA format)
1	Leader-Flag-C4-LC-scFvC4	MNFGFSLIFLVLVLKGVQCEVKLVPR**QL**DYKDDDDK**EL***DIVMTQ SHKFMSTSVGDRVSITCKASQDVDTAVAWYQQKPGQSPKLLIYW ASTRHTGVPDRFTGSGSGTDYTLTISSVQAEDLARYYCQQYYSV PPTFGGGTKL***GS**EIKRTVAAPSVFIFPPSDEQLKSGTASVVCLL NNFYPREAKVQWKVDNALQSGNSQESVTEQDSKDSTYSLSSTLT

		LSKADYEKHKVYACEVTHQGLSSPVTKSFNRGEC**LE**QVQLLQSGPELVKPGASVKLSCKASGYSFTSYDINWVKQRPGQGLEWVGWIYPRDGDTKYNEKFKGKAILTVDTSSNTAYMNLHSLTSEDSAVYFCARLTGPYWYFDVWGTGTTVTVSS**RSSTKGPKLEEGEFSEAQL**DIVMTQSHKFMSTSVGDRVSITCKASQDVDTAVAWYQQKPGQSPKLLIYWASTRHTGVPDRFTGSGSGTDYTLTISSVQAEDLARYYCQQYYSVPPTFGGGTKLEIK
2	Leader-Flag-C4-Fab1	MNFGFSLIFLVLVLKGVQCEVKLVPR**QL**DYKDDDDK**EL**QVQLLQSGPELVKPGASVKLSCKASGYSFTSYDINWVKQRPGQGLEWVGWIYPRDGDTKYNEKFKGKAILTVDTSSNTAYMNLHSLTSEDSAVYFCARLTGPYWYFDVWGTGTTVTVSS**GS**SSASTKGPSVFPLAPSSKSTSGGTAALGCLVKDYFPEPVTVSWNSGALTSGVHTFPAVLQSSGLYSLSSVVTVPSSSLGTQTYICNVNHKPSNTKVDKKVEPKSCDKTHT
3	Leader-C4-Fab2	MNFGFSLIFLVLVLKGVQCEVKLVPR**QL**QVQLLQSGPELVKPGASVKLSCKASGYSFTSYDINWVKQRPGQGLEWVGWIYPRDGDTKYNEKFKGKAILTVDTSSNTAYMNLHSLTSEDSAVYFCARLTGPYWYFDVWGTGTTVTVSS**GS**SSASTKGPSVFPLAPSSKSTSGGTAALGCLVKDYFPEPVTVSWNSGALTSGVHTFPAVLQSSGLYSLSSVVTVPSSSLGTQTYICNVNHKPSNTKVDKKVEPKSCDKTHTCPPCPA
4)	Leader-Flag-C4-LC	MNFGFSLIFLVLVLKGVQCEVKLVPR**QL**DYKDDDDK**EL**DIVMTQSHKFMSTSVGDRVSITCKASQDVDTAVAWYQQKPGQSPKLLIYWASTRHTGVPDRFTGSGSGTDYTLTISSVQAEDLARYYCQQYYSVPPTFGGGTKL**GS**EIKRTVAAPSVFIFPPSDEQLKSGTASVVCLLNNFYPREAKVQWKVDNALQSGNSQESVTEQDSKDSTYSLSSTLTLSKADYEKHKVYACEVTHQGLSSPVTKSFNRGEC
5	Leader-Flag-C4-IgG1(N297A)-scFvC4	MNFGFSLIFLVLVLKGVQCEVKLVPR**QL**DYKDDDDK**EL**QVQLLQSGPELVKPGASVKLSCKASGYSFTSYDINWVKQRPGQGLEWVGWIYPRDGDTKYNEKFKGKAILTVDTSSNTAYMNLHSLTSEDSAVYFCARLTGPYWYFDVWGTGTTVTVSS**GS**SSASTKGPSVFPLAPSSKSTSGGTAALGCLVKDYFPEPVTVSWNSGALTSGVHTFPAVLQSSGLYSLSSVVTVPSSSLGTQTYICNVNHKPSNTKVDKKVEPKSCDKTHTCPPCPAPELLGGPSVFLFPPKPKDTLMISRTPEVTCVVVDVSHEDPEVKFNWYVDGVEVHNAKTKPREEQYASTYRVVSVLTVLHQDWLNGKEYKCKVSNKALPAPIEKTISKAKGQPREPQVYTLPPSRDELTKNQVSLTCLVKGFYPSDIAVEWESNGQPENNYKTTPPVLDSDGSFFLYSKLTVDKSRWQQGNVFSCSVMHEALHNHYTQKSLSLSPGK**EFLE**QVQLLQSGPELVKPGASVKLSCKASGYSFTSYDINWVKQRPGQGLEWVGWIYPRDGDTKYNEKFKGKAILTVDTSSNTAYMNLHSLTSEDSAVYFCARLTGPYWYFDVWGTGTTVTVSS**RSSTKGPKLEEGEFSEAQL**DIVMTQSHKFMSTSVGDRVSITCKASQDVDTAVAWYQQKPGQSPKLLIYWASTRHTGVPDRFTGSGSGTDYTLTISSVQAEDLARYYCQQYYSVPPTFGGGTKLEIK
6	Leader-Flag-scFvC4-CL	MNFGFSLIFLVLVLKGVQCEVKLVPR**QL**DYKDDDDK**EF**QVQLLQSGPELVKPGASVKLSCKASGYSFTSYDINWVKQRPGQGLEWVGWIYPRDGDTKYNEKFKGKAILTVDTSSNTAYMNLHSLTSEDSAVYFCARLTGPYWYFDVWGTGTTVTVSS**RSSTKGPKLEEGEFSEAQL**DIVMTQSHKFMSTSVGDRVSITCKASQDVDTAVAWYQQKPGQSPKLLIYWASTRHTGVPDRFTGSGSGTDYTLTISSVQAEDLARYYCQQYYSVPPTFGGGTKLEIK**GS**EIKRTVAAPSVFIFPPSDEQLKSGTASVVCLLNNFYPREAKVQWKVDNALQSGNSQESVTEQDSKDSTYSLSSTLTLSKADYEKHKVYACEVTHQGLSSPVTKSFNRGEC
7	Leader-Flag-scFvC4-CH1/CH2/CH3(N297A)-scFvC4	MNFGFSLIFLVLVLKGVQCEVKLVPR**QL**DYKDDDDK**EF**QVQLLQSGPELVKPGASVKLSCKASGYSFTSYDINWVKQRPGQGLEWVGWIYPRDGDTKYNEKFKGKAILTVDTSSNTAYMNLHSLTSEDSAVYFCARLTGPYWYFDVWGTGTTVTVSS**RSSTKGPKLEEGEFSEAQL**DIVMTQSHKFMSTSVGDRVSITCKASQDVDTAVAWYQQKPGQSPKLLIYWASTRHTGVPDRFTGSGSGTDYTLTISSVQAEDLARYYCQQYYSVPPTFGGGTKLEIK**GS**SSASTKGPSVFPLAPSSKSTSGGTAALGCLVKDYFPEPVTVSWNSGALTSGVHTFPAVLQSSGLYSLSSVVTVPSSSLGTQTYICNVNHKPSNTKVDKKVEPKSCDKTHTC

		PPCPAPELLGGPSVFLFPPKPKDTLMISRTPEVTCVVVDVSHED<br>PEVKFNWYVDGVEVHNAKTKPREEQYASTYRVVSVLTVLHQDWL<br>NGKEYKCKVSNKALPAPIEKTISKAKGQPREPQVYTLPPSRDEL<br>TKNQVSLTCLVKGFYPSDIAVEWESNGQPENNYKTTPPVLDSDG<br>SFFLYSKLTVDKSRWQQGNVFSCSVMHEALHNHYTQKSLSLSPG<br>KLEQVQLLQSGPELVKPGASVKLSCKASGYSFTSYDINWVKQRP<br>GQGLEWVGWIYPRDGDTKYNEKFKGKAILTVDTSSNTAYMNLHS<br>LTSEDSAVYFCARLTGPYWYFDVWGTGTTVTVSSRSSTKGPKLE<br>EGEFSEAQLDIVMTQSHKFMSTSVGDRVSITCKASQDVDTAVAW<br>YQQKPGQSPKLLIYWASTRHTGVPDRFTGSGSGTDYTLTISSVQ<br>AEDLARYYCQQYYSVPPTFGGGTKLEIK
8	Leader-Flag-scFvC4-CL-scFvC4	MNFGFSLIFLVLVLKGVQCEVKLVPRQLDYKDDDDKEFQVQLLQ<br>SGPELVKPGASVKLSCKASGYSFTSYDINWVKQRPGQGLEWVGW<br>IYPRDGDTKYNEKFKGKAILTVDTSSNTAYMNLHSLTSEDSAVY<br>FCARLTGPYWYFDVWGTGTTVTVSSRSSTKGPKLEEGEFSEAQL<br>DIVMTQSHKFMSTSVGDRVSITCKASQDVDTAVAWYQQKPGQSP<br>KLLIYWASTRHTGVPDRFTGSGSGTDYTLTISSVQAEDLARYYC<br>QQYYSVPPTFGGGTKLEIKGSEIKRTVAAPSVFIFPPSDEQLKS<br>GTASVVCLLNNFYPREAKVQWKVDXALQSGNSQESVTEQDSKDS<br>TYSLSSTLTLSKADYEKHKVYACEVTHQGLSSPVTKSFNRGECL<br>EQVQLLQSGPELVKPGASVKLSCKASGYSFTSYDINWVKQRPGQ<br>GLEWVGWIYPRDGDTKYNEKFKGKAILTVDTSSNTAYMNLHSLT<br>SEDSAVYFCARLTGPYWYFDVWGTGTTVTVSSRSSTKGPKLEEG<br>EFSEAQLDIVMTQSHKFMSTSVGDRVSITCKASQDVDTAVAWYQ<br>QKPGQSPKLLIYWASTRHTGVPDRFTGSGSGTDYTLTISSVQAE<br>DLARYYCQQYYSVPPTFGGGTKLEIK
9	Leader-Flag-scFvC4-CH1/CH2/CH3(N297A)	MNFGFSLIFLVLVLKGVQCEVKLVPRQLDYKDDDDKEFQVQLLQ<br>SGPELVKPGASVKLSCKASGYSFTSYDINWVKQRPGQGLEWVGW<br>IYPRDGDTKYNEKFKGKAILTVDTSSNTAYMNLHSLTSEDSAVY<br>FCARLTGPYWYFDVWGTGTTVTVSSRSSTKGPKLEEGEFSEAQL<br>DIVMTQSHKFMSTSVGDRVSITCKASQDVDTAVAWYQQKPGQSP<br>KLLIYWASTRHTGVPDRFTGSGSGTDYTLTISSVQAEDLARYYC<br>QQYYSVPPTFGGGTKLEIKGSSSASTKGPSVFPLAPSSKSTSGG<br>TAALGCLVKDYFPEPVTVSWNSGALTSGVHTFPAVLQSSGLYSL<br>SSVVTVPSSSLGTQTYICNVNHKPSNTKVDKKVEPKSCDKTHTC<br>PPCPAPELLGGPSVFLFPPKPKDTLMISRTPEVTCVVVDVSHED<br>PEVKFNWYVDGVEVHNAKTKPREEQYASTYRVVSVLTVLHQDWL<br>NGKEYKCKVSNKALPAPIEKTISKAKGQPREPQVYTLPPSRDEL<br>TKNQVSLTCLVKGFYPSDIAVEWESNGQPENNYKTTPPVLDSDG<br>SFFLYSKLTVDKSRWQQGNVFSCSVMHEALHNHYTQKSLSLSPG<br>K
10	Leader-Flag-C4-IgG1(N297A)-TNCscFvC4	MNFGFSLIFLVLVLKGVQCEVKLVPRQLDYKDDDDKELQVQLLQ<br>SGPELVKPGASVKLSCKASGYSFTSYDINWVKQRPGQGLEWVGW<br>IYPRDGDTKYNEKFKGKAILTVDTSSNTAYMNLHSLTSEDSAVY<br>FCARLTGPYWYFDVWGTGTTVTVSSGSSSASTKGPSVFPLAPSS<br>KSTSGGTAALGCLVKDYFPEPVTVSWNSGALTSGVHTFPAVLQS<br>SGLYSLSSVVTVPSSSLGTQTYICNVNHKPSNTKVDKKVEPKSC<br>DKTHTCPPCPAPELLGGPSVFLFPPKPKDTLMISRTPEVTCVVV<br>DVSHEDPEVKFNWYVDGVEVHNAKTKPREEQYASTYRVVSVLTV<br>LHQDWLNGKEYKCKVSNKALPAPIEKTISKAKGQPREPQVYTLP<br>PSRDELTKNQVSLTCLVKGFYPSDIAVEWESNGQPENNYKTTPP<br>VLDSDGSFFLYSKLTVDKSRWQQGNVFSCSVMHEALHNHYTQKS<br>LSLSPGKEFLEDIACGCAAAPDIKDLLSRLEELEGLVSSLREQG<br>TGGGSVEQVQLLQSGPELVKPGASVKLSCKASGYSFTSYDINWV<br>KQRPGQGLEWVGWIYPRDGDTKYNEKFKGKAILTVDTSSNTAYM<br>NLHSLTSEDSAVYFCARLTGPYWYFDVWGTGTTVTVSSRSSTKG<br>PKLEEGEFSEAQLDIVMTQSHKFMSTSVGDRVSITCKASQDVDT<br>AVAWYQQKPGQSPKLLIYWASTRHTGVPDRFTGSGSGTDYTLTI<br>SSVQAEDLARYYCQQYYSVPPTFGGGTKLEIK
11	Leader-Flag-C4-IgG1(N297A)-TNC	MNFGFSLIFLVLVLKGVQCEVKLVPRQLDYKDDDDKELQVQLLQ<br>SGPELVKPGASVKLSCKASGYSFTSYDINWVKQRPGQGLEWVGW<br>IYPRDGDTKYNEKFKGKAILTVDTSSNTAYMNLHSLTSEDSAVY

		FCARLTGPYWYFDVWGTGTTVTVSS**GS**SSASTKGPSVFPLAPSS<br>KSTSGGTAALGCLVKDYFPEPVTVSWNSGALTSGVHTFPAVLQS<br>SGLYSLSSVVTVPSSSLGTQTYICNVNHKPSNTKVDKKVEPKSC<br>DKTHTCPPCPAPELLGGPSVFLFPPKPKDTLMISRTPEVTCVVV<br>DVSHEDPEVKFNWYVDGVEVHNAKTKPREEQYASTYRVVSVLTV<br>LHQDWLNGKEYKCKVSNKALPAPIEKTISKAKGQPREPQVYTLP<br>PSRDELTKNQVSLTCLVKGFYPSDIAVEWESNGQPENNYKTTPP<br>VLDSDGSFFLYSKLTVDKSRWQQGNVFSCSVMHEALHNHYTQKS<br>LSLSPGK**EFLE**DIACGCAAAPDIKDLLSRLEELEGLVSSLREQG<br>TG
12	Leader-Flag-scFvC4-TNC-Fc(DANA)	MNFGFSLIFLVLVLKGVQCEVKLVPR**QL**DYKDDDDK**EF**QVQLLQ<br>SGPELVKPGASVKLSCKASGYSFTSYDINWVKQRPGQGLEWVGW<br>IYPRDGDTKYNEKFKGKAILTVDTSSNTAYMNLHSLTSEDSAVY<br>FCARLTGPYWYFDVWGTGTTVTVSS**RSSTKGPKLEEGEFSEAQL**<br>DIVMTQSHKFMSTSVGDRVSITCKASQDVDTAVAWYQQKPGQSP<br>KLLIYWASTRHTGVPDRFTGSGSGTDYTLTISSVQAEDLARYYC<br>QQYYSVPPTFGGGTKLEIK**GS**DIACGCAAAPDIKDLLSRLEELE<br>GLVSSLREQGTG**EF**KTHTCPPCPAPELLGGPSVFLFPPKPKDTL<br>MISRTPEVTCVVVAVSHEDPEVKFNWYVDGVEVHNAKTKPREEQ<br>YASTYRVVSVLTVLHQDWLNGKEYKCKVSNKALPAPIEKTISKA<br>KGQPREPQVYTLPPSRDELTKNQVSLTCLVKGFYPSDIAVEWES<br>NGQPENNYKTTPPVLDSDGSFFLYSKLTVDKSRWQQGNVFSCSV<br>MHEALHNHYTQKSLSLSPGK**LE**
13	Leader-Flag-scFvC4-Fc(DANA)-TNC	MNFGFSLIFLVLVLKGVQCEVKLVPR**QL**DYKDDDDK**EF**QVQLLQ<br>SGPELVKPGASVKLSCKASGYSFTSYDINWVKQRPGQGLEWVGW<br>IYPRDGDTKYNEKFKGKAILTVDTSSNTAYMNLHSLTSEDSAVY<br>FCARLTGPYWYFDVWGTGTTVTVSS**RSSTKGPKLEEGEFSEAQL**<br>DIVMTQSHKFMSTSVGDRVSITCKASQDVDTAVAWYQQKPGQSP<br>KLLIYWASTRHTGVPDRFTGSGSGTDYTLTISSVQAEDLARYYC<br>QQYYSVPPTFGGGTKLEIK**GS**KTHTCPPCPAPELLGGPSVFLFP<br>PKPKDTLMISRTPEVTCVVVAVSHEDPEVKFNWYVDGVEVHNAK<br>TKPREEQYASTYRVVSVLTVLHQDWLNGKEYKCKVSNKALPAPI<br>EKTISKAKGQPREPQVYTLPPSRDELTKNQVSLTCLVKGFYPSD<br>IAVEWESNGQPENNYKTTPPVLDSDGSFFLYSKLTVDKSRWQQG<br>NVFSCSVMHEALHNHYTQKSLSLSPGK**LE**DIACGCAAAPDIKDL<br>LSRLEELEGLVSSLREQGTG
14	Leader-Flag-C4-IgG1(N297A-RGY)	MNFGFSLIFLVLVLKGVQCEVKLVPR**QL**DYKDDDDK**EL**QVQLLQ<br>SGPELVKPGASVKLSCKASGYSFTSYDINWVKQRPGQGLEWVGW<br>IYPRDGDTKYNEKFKGKAILTVDTSSNTAYMNLHSLTSEDSAVY<br>FCARLTGPYWYFDVWGTGTTVTVSS**GS**SSASTKGPSVFPLAPSS<br>KSTSGGTAALGCLVKDYFPEPVTVSWNSGALTSGVHTFPAVLQS<br>SGLYSLSSVVTVPSSSLGTQTYICNVNHKPSNTKVDKKVEPKSC<br>DKTHTCPPCPAPELLGGPSVFLFPPKPKDTLMISRTPEVTCVVV<br>DVSHEDPEVKFNWYVDGVEVHNAKTKPREEQYASTYRVVSVLTV<br>LHQDWLNGKEYKCKVSNKALPAPIEKTISKAKGQPRRPQVYTLP<br>PSRDELTKNQVSLTCLVKGFYPSDIAVEWESNGQPENNYKTTPP<br>VLDSDGSFFLYSKLTVDKSRWQQGNVFSCSVMHGALHNHYTQKY<br>LSLSPGK
15	Leader-Flag-scFvC4-CH1/CH2/CH3(N297A)-TNC	MNFGFSLIFLVLVLKGVQCEVKLVPR**QL**DYKDDDDK**EF**QVQLLQ<br>SGPELVKPGASVKLSCKASGYSFTSYDINWVKQRPGQGLEWVGW<br>IYPRDGDTKYNEKFKGKAILTVDTSSNTAYMNLHSLTSEDSAVY<br>FCARLTGPYWYFDVWGTGTTVTVSS**RSSTKGPKLEEGEFSEAQL**<br>DIVMTQSHKFMSTSVGDRVSITCKASQDVDTAVAWYQQKPGQSP<br>KLLIYWASTRHTGVPDRFTGSGSGTDYTLTISSVQAEDLARYYC<br>QQYYSVPPTFGGGTKLEIK**GS**SSASTKGPSVFPLAPSSKSTSGG<br>TAALGCLVKDYFPEPVTVSWNSGALTSGVHTFPAVLQSSGLYSL<br>SSVVTVPSSSLGTQTYICNVNHKPSNTKVDKKVEPKSCDKTHTC<br>PPCPAPELLGGPSVFLFPPKPKDTLMISRTPEVTCVVVDVSHED<br>PEVKFNWYVDGVEVHNAKTKPREEQYASTYRVVSVLTVLHQDWL<br>NGKEYKCKVSNKALPAPIEKTISKAKGQPREPQVYTLPPSRDEL<br>TKNQVSLTCLVKGFYPSDIAVEWESNGQPENNYKTTPPVLDSDG<br>SFFLYSKLTVDKSRWQQGNVFSCSVMHEALHNHYTQKSLSLSPG

		KLEDIACGCAAAPDIKDLLSRLEELEGLVSSLREQGTG
16	Leader-Flag-C9-CL	MNFGFSLIFLVLVLKGVQCEVKLVPRQLDYKDDDDKELQIVLTQ<br>SPAIMSASPGEKVTITCSASSSVSYMHWFQQKPGTSPKLWIYST<br>SNLASGVPARFSGSGSGTSYSLTISRMEAEDAATYYCQQRSSYP<br>PTFGGGTKLEIKGSEIKRTVAAPSVFIFPPSDEQLKSGTASVVC<br>LLNNFYPREAKVQWKVDNALQSGNSQESVTEQDSKDSTYSLSST<br>LTLSKADYEKHKVYACEVTHQGLSSPVTKSFNRGEC
17	Leader-Flag-C9-IgG1(N297A)	MNFGFSLIFLVLVLKGVQCEVKLVPRQLDYKDDDDKEFQIQLVQ<br>SGPELKKPGETVKISCKASGYTFTTAGMQWVQKMPGKGFKWIGW<br>INTHSGEPKYAEDFKGRFAFSLETSASTAYLQISNLKNEDTATY<br>FCARWDGTGYWGQGTTLTVSSRSSSASTKGPSVFPLAPSSKSTS<br>GGTAALGCLVKDYFPEPVTVSWNSGALTSGVHTFPAVLQSSGLY<br>SLSSVVTVPSSSLGTQTYICNVNHKPSNTKVDKKVEPKSCDKTH<br>TCPPCPAPELLGGPSVFLFPPKPKDTLMISRTPEVTCVVVDVSH<br>EDPEVKFNWYVDGVEVHNAKTKPREEQYASTYRVVSVLTVLHQD<br>WLNGKEYKCKVSNKALPAPIEKTISKAKGQPREPQVYTLPPSRD<br>ELTKNQVSLTCLVKGFYPSDIAVEWESNGQPENNYKTTPPVLDS<br>DGSFFLYSKLTVDKSRWQQGNVFSCSVMHEALHNHYTQKSLSLS<br>PGK
18	Leader-Flag-C9-IgG1(N297A)-scFvC9	MNFGFSLIFLVLVLKGVQCEVKLVPRQLDYKDDDDKEFQIQLVQ<br>SGPELKKPGETVKISCKASGYTFTTAGMQWVQKMPGKGFKWIGW<br>INTHSGEPKYAEDFKGRFAFSLETSASTAYLQISNLKNEDTATY<br>FCARWDGTGYWGQGTTLTVSSRSSSASTKGPSVFPLAPSSKSTS<br>GGTAALGCLVKDYFPEPVTVSWNSGALTSGVHTFPAVLQSSGLY<br>SLSSVVTVPSSSLGTQTYICNVNHKPSNTKVDKKVEPKSCDKTH<br>TCPPCPAPELLGGPSVFLFPPKPKDTLMISRTPEVTCVVVDVSH<br>EDPEVKFNWYVDGVEVHNAKTKPREEQYASTYRVVSVLTVLHQD<br>WLNGKEYKCKVSNKALPAPIEKTISKAKGQPREPQVYTLPPSRD<br>ELTKNQVSLTCLVKGFYPSDIAVEWESNGQPENNYKTTPPVLDS<br>DGSFFLYSKLTVDKSRWQQGNVFSCSVMHEALHNHYTQKSLSLS<br>PGKLEQIQLVQSGPELKKPGETVKISCKASGYTFTTAGMQWVQK<br>MPGKGFKWIGWINTHSGEPKYAEDFKGRFAFSLETSASTAYLQI<br>SNLKNEDTATYFCARWDGTGYWGQGTTLTVSSRSSTKGPKLEEG<br>EFSEAQLQIVLTQSPAIMSASPGEKVTITCSASSSVSYMHWFQQ<br>KPGTSPKLWIYSTSNLASGVPARFSGSGSGTSYSLTISRMEAED<br>AATYYCQQRSSYPPTFGGGTKLEIK
19	Leader-Flag-scFvC9-CL	MNFGFSLIFLVLVLKGVQCEVKLVPRQLDYKDDDDKEFQIQLVQ<br>SGPELKKPGETVKISCKASGYTFTTAGMQWVQKMPGKGFKWIGW<br>INTHSGEPKYAEDFKGRFAFSLETSASTAYLQISNLKNEDTATY<br>FCARWDGTGYWGQGTTLTVSSRSSTKGPKLEEGEFSEAQLQIVL<br>TQSPAIMSASPGEKVTITCSASSSVSYMHWFQQKPGTSPKLWIY<br>STSNLASGVPARFSGSGSGTSYSLTISRMEAEDAATYYCQQRSS<br>YPPTFGGGTKLEIKGSEIKRTVAAPSVFIFPPSDEQLKSGTASV<br>VCLLNNFYPREAKVQWKVDNALQSGNSQESVTEQDSKDSTYSLS<br>STLTLSKADYEKHKVYACEVTHQGLSSPVTKSFNRGEC
20	Leader-Flag-scFvC9-CH1/CH2/CH3(N297A)	MNFGFSLIFLVLVLKGVQCEVKLVPRQLDYKDDDDKEFQIQLVQ<br>SGPELKKPGETVKISCKASGYTFTTAGMQWVQKMPGKGFKWIGW<br>INTHSGEPKYAEDFKGRFAFSLETSASTAYLQISNLKNEDTATY<br>FCARWDGTGYWGQGTTLTVSSRSSTKGPKLEEGEFSEAQLQIVL<br>TQSPAIMSASPGEKVTITCSASSSVSYMHWFQQKPGTSPKLWIY<br>STSNLASGVPARFSGSGSGTSYSLTISRMEAEDAATYYCQQRSS<br>YPPTFGGGTKLEIKGSSSASTKGPSVFPLAPSSKSTSGGTAALG<br>CLVKDYFPEPVTVSWNSGALTSGVHTFPAVLQSSGLYSLSSVVT<br>VPSSSLGTQTYICNVNHKPSNTKVDKKVEPKSCDKTHTCPPCPA<br>PELLGGPSVFLFPPKPKDTLMISRTPEVTCVVVDVSHEDPEVKF<br>NWYVDGVEVHNAKTKPREEQYASTYRVVSVLTVLHQDWLNGKEY<br>KCKVSNKALPAPIEKTISKAKGQPREPQVYTLPPSRDELTKNQV<br>SLTCLVKGFYPSDIAVEWESNGQPENNYKTTPPVLDSDGSFFLY<br>SKLTVDKSRWQQGNVFSCSVMHEALHNHYTQKSLSLSPGK
21	Leader-Flag-C9-IgG1(N297A)-TNC	MNFGFSLIFLVLVLKGVQCEVKLVPRQLDYKDDDDKEFQIQLVQ<br>SGPELKKPGETVKISCKASGYTFTTAGMQWVQKMPGKGFKWIGW<br>INTHSGEPKYAEDFKGRFAFSLETSASTAYLQISNLKNEDTATY

		*FCARWDGTGYWGQGTTLTVSS***RS**SSASTKGPSVFPLAPSSKSTS<br>GGTAALGCLVKDYFPEPVTVSWNSGALTSGVHTFPAVLQSSGLY<br>SLSSVVTVPSSSLGTQTYICNVNHKPSNTKVDKKVEPKSCDKTH<br>TCPPCPAPELLGGPSVFLFPPKPKDTLMISRTPEVTCVVVDVSH<br>EDPEVKFNWYVDGVEVHNAKTKPREEQYASTYRVVSVLTVLHQD<br>WLNGKEYKCKVSNKALPAPIEKTISKAKGQPREPQVYTLPPSRD<br>ELTKNQVSLTCLVKGFYPSDIAVEWESNGQPENNYKTTPPVLDS<br>DGSFFLYSKLTVDKSRWQQGNVFSCSVMHEALHNHYTQKSLSLS<br>PGK**LE***DIACGCAAAPDIKDLLSRLEELEGLVSSLREQGTG*
22	Leader-Flag-C19-CL	MNFGFSLIFLVLVLKGVQCEVKLVPR**QL**DYKDDDDK**EL***DIVMAQ<br>SQKFMSVSVGDRVSVTCKASQNVGTNVAWYQQRPGQSPKALIYS<br>ASYRYSGVPDRFTGSGSGTDFTLTISNVQSEDLAEYFCQQFDSH<br>PLTFGAGTKLELK***GS**EIKRTVAAPSVFIFPPSDEQLKSGTASVV<br>CLLNNFYPREAKVQWKVDNALQSGNSQESVTEQDSKDSTYSLSS<br>TLTLSKADYEKHKVYACEVTHQGLSSPVTKSFNRGEC
23	Leader-C19-IgG1(N297A)	MNFGFSLIFLVLVLKGVQCEVKLVPR**QL***QIQLVQSGPEVKKPGE<br>TVKISCKASGYTFTIHGMSWVKQAPGKGLKWMGWINTYSGVPTY<br>ANDFKGRFAFSLETSASTAYLQINNLKNEDTATYFCARDEVRRG<br>FGFAYWGQGTLVTVSA***GS**SSASTKGPSVFPLAPSSKSTSGGTAA<br>LGCLVKDYFPEPVTVSWNSGALTSGVHTFPAVLQSSGLYSLSSV<br>VTVPSSSLGTQTYICNVNHKPSNTKVDKKVEPKSCDKTHTCPPC<br>PAPELLGGPSVFLFPPXPKDTLMISRTPEVTCVVVDVSHEDPEV<br>KFNWYVDGVEVHNAKTKPREEQYASTYRVVSVLTVLHQDWLNGK<br>EYKCKVSNKALPAPIEKTISKAKGQPREPQVYTLPPSRDELTKN<br>QVSLTCLVKGFYPSDIAVEWESNGQPENNYKTTPPVLDSDGSFF<br>LYSKLTVDKSRWQQGNVFSCSVMHEALHNHYTQKSLSLSPGK
24	Leader-Flag-C19-IgG1(N297A)-scFvC19	MNFGFSLIFLVLVLKGVQCEVKLVPR**QL**DYKDDDDK**EL***QIQLVQ<br>SGPEVKKPGETVKISCKASGYTFTIHGMSWVKQAPGKGLKWMGW<br>INTYSGVPTYANDFKGRFAFSLETSASTAYLQINNLKNEDTATY<br>FCARDEVRRGFGFAYWGQGTLVTVSA***GS**SSASTKGPSVFPLAPS<br>SKSTSGGTAALGCLVKDYFPEPVTVSWNSGALTSGVHTFPAVLQ<br>SSGLYSLSSVVTVPSSSLGTQTYICNVNHKPSNTKVDKKVEPKS<br>CDKTHTCPPCPAPELLGGPSVFLFPPKPKDTLMISRTPEVTCVV<br>VDVSHEDPEVKFNWYVDGVEVHNAKTKPREEQYASTYRVVSVLT<br>VLHQDWLNGKEYKCKVSNKALPAPIEKTISKAKGQPREPQVYTL<br>PPSRDELTKNQVSLTCLVKGFYPSDIAVEWESNGQPENNYKTTP<br>PVLDSDGSFFLYSKLTVDKSRWQQGNVFSCSVMHEALHNHYTQK<br>SLSLSPGK**LE***QIQLVQSGPEVKKPGETVKISCKASGYTFTIHGM<br>SWVKQAPGKGLKWMGWINTYSGVPTYANDFKGRFAFSLETSAST<br>AYLQINNLKNEDTATYFCARDEVRRGFGFAYWGQGTLVTVSA***RS**<br>***STKGPKLEEGEFSEA*QL***DIVMAQSQKFMSVSVGDRVSVTCKASQ<br>NVGTNVAWYQQRPGQSPKALIYSASYRYSGVPDRFTGSGSGTDF<br>TLTISNVQSEDLAEYFCQQFDSHPLTFGAGTKLELK*
25	Leader-Flag-scFvC19-CL	MNFGFSLIFLVLVLKGVQCEVKLVPR**QL**DYKDDDDK**EF***QIQLVQ<br>SGPEVKKPGETVKISCKASGYTFTIHGMSWVKQAPGKGLKWMGW<br>INTYSGVPTYANDFKGRFAFSLETSASTAYLQINNLKNEDTATY<br>FCARDEVRRGFGFAYWGQGTLVTVSA***RS*STKGPKLEEGEFSEA*Q<br>L***DIVMAQSQKFMSVSVGDRVSVTCKASQNVGTNVAWYQQRPGQS<br>PKALIYSASYRYSGVPDRFTGSGSGTDFTLTISNVQSEDLAEYF<br>CQQFDSHPLTFGAGTKLELK***GS**EIKRTVAAPSVFIFPPSDEQLK<br>SGTASVVCLLNNFYPREAKVQWKVDNALQSGNSQESVTEQDSKD<br>STYSLSSTLTLSKADYEKHKVYACEVTHQGLSSPVTKSFNRGEC
26	Leader-Flag-scFvC19-CH1/CH2/CH3(N297A)	MNFGFSLIFLVLVLKGVQCEVKLVPR**QL**DYKDDDDK**EF***QIQLVQ<br>SGPEVKKPGETVKISCKASGYTFTIHGMSWVKQAPGKGLKWMGW<br>INTYSGVPTYANDFKGRFAFSLETSASTAYLQINNLKNEDTATY<br>FCARDEVRRGFGFAYWGQGTLVTVSA***RS*STKGPKLEEGEFSEA*Q<br>L***DIVMAQSQKFMSVSVGDRVSVTCKASQNVGTNVAWYQQRPGQS<br>PKALIYSASYRYSGVPDRFTGSGSGTDFTLTISNVQSEDLAEYF<br>CQQFDSHPLTFGAGTKLELK***GS**SSASTKGPSVFPLAPSSKSTSG<br>GTAALGCLVKDYFPEPVTVSWNSGALTSGVHTFPAVLQSSGLYS<br>LSSVVTVPSSSLGTQTYICNVNHKPSNTKVDKKVEPKSCDKTHT<br>CPPCPAPELLGGPSVFLFPPKPKDTLMISRTPEVTCVVVDVSHE

		DPEVKFNWYVDGVEVHNAKTKPREEQYASTYRVVSVLTVLHQDW<br>LNGKEYKCKVSNKALPAPIEKTISKAKGQPREPQVYTLPPSRDE<br>LTKNQVSLTCLVKGFYPSDIAVEWESNGQPENNYKTTPPVLDSD<br>GSFFLYSKLTVDKSRWQQGNVFSCSVMHEALHNHYTQKSLSLSP<br>GK
27	Leader-Flag-C19-IgG1(N297A)-TNC	MNFGFSLIFLVLVLKGVQCEVKLVPR**QL**DYKDDDDK**EF***QIQLVQ*<br>*SGPEVKKPGETVKISCKASGYTFTIHGMSWVKQAPGKGLKWMGW*<br>*INTYSGVPTYANDFKGRFAFSLETSASTAYLQINNLKNEDTATY*<br>*FCARDEVRRGFGFAYWGQGTLVTVSA***RS**SSASTKGPSVFPLAPS<br>SKSTSGGTAALGCLVKDYFPEPVTVSWNSGALTSGVHTFPAVLQ<br>SSGLYSLSSVVTVPSSSLGTQTYICNVNHKPSNTKVDKKVEPKS<br>CDKTHTCPPCPAPELLGGPSVFLFPPKPKDTLMISRTPEVTCVV<br>VDVSHEDPEVKFNWYVDGVEVHNAKTKPREEQYASTYRVVSVLT<br>VLHQDWLNGKEYKCKVSNKALPAPIEKTISKAKGQPREPQVYTL<br>PPSRDELTKNQVSLTCLVKGFYPSDIAVEWESNGQPENNYKTTP<br>PVLDSDGSFFLYSKLTVDKSRWQQGNVFSCSVMHEALHNHYTQK<br>SLSLSPGK**LE***DIACGCAAAPDIKDLLSRLEELEGLVSSLREQGT*<br>*G*
28	Leader-Flag-C40-CL	MNFGFSLIFLVLVLKGVQCEVKLVPR**QL**DYKDDDDK**EL***DIQMTQ*<br>*SPSSLSASLGGKVTITCKASQDINKFIAWYQHKPGKGPRLLIHY*<br>*TSTLQPGIPSRFSGSGSGRDYSFSISNLEPEDIATYYCLQYDNL*<br>*YTFGGGTKLEIK***GS**EIKRTVAAPSVFIFPPSDEQLKSGTASVVC<br>LLNNFYPREAKVQWKVDNALQSGNSQESVTEQDSKDSTYSLSST<br>LTLSKADYEKHKVYACEVTHQGLSSPVTKSFNRGEC
29	Leader-Flag-C40-IgG1(N297A)	MNFGFSLIFLVLVLKGVQCEVKLVPR**QL**DYKDDDDK**EF***QVTLKE*<br>*SGPGILQPSQTLSLTCSFSGFSLSTFGMGVGWIRQPSGKGLEWL*<br>*AHIWWDDDKYYNPALKSRLTISKDTSENQVFLKIANVDTADTAT*<br>*YYCVRIAGTRYFDVWGTGTTVTVSS***RS**SSASTKGPSVFPLAPSS<br>KSTSGGTAALGCLVKDYFPEPVTVSWNSGALTSGVHTFPAVLQS<br>SGLYSLSSVVTVPSSSLGTQTYICNVNHKPSNTKVDKKVEPKSC<br>DKTHTCPPCPAPELLGGPSVFLFPPKPKDTLMISRTPEVTCVVV<br>DVSHEDPEVKFNWYVDGVEVHNAKTKPREEQYASTYRVVSVLTV<br>LHQDWLNGKEYKCKVSNKALPAPIEKTISKAKGQPREPQVYTLP<br>PSRDELTKNQVSLTCLVKGFYPSDIAVEWESNGQPENNYKTTPP<br>VLDSDGSFFLYSKLTVDKSRWQQGNVFSCSVMHEALHNHYTQKS<br>LSLSPGK
30	Leader-Flag-C40-IgG1(N297A)-scFvC40	MNFGFSLIFLVLVLKGVQCEVKLVPR**QL**DYKDDDDK**EF***QVTLKE*<br>*SGPGILQPSQTLSLTCSFSGFSLSTFGMGVGWIRQPSGKGLEWL*<br>*AHIWWDDDKYYNPALKSRLTISKDTSENQVFLKIANVDTADTAT*<br>*YYCVRIAGTRYFDVWGTGTTVTVSS***RS**SSASTKGPSVFPLAPSS<br>KSTSGGTAALGCLVKDYFPEPVTVSWNSGALTSGVHTFPAVLQS<br>SGLYSLSSVVTVPSSSLGTQTYICNVNHKPSNTKVDKKVEPKSC<br>DKTHTCPPCPAPELLGGPSVFLFPPKPKDTLMISRTPEVTCVVV<br>DVSHEDPEVKFNWYVDGVEVHNAKTKPREEQYASTYRVVSVLTV<br>LHQDWLNGKEYKCKVSNKALPAPIEKTISKAKGQPREPQVYTLP<br>PSRDELTKNQVSLTCLVKGFYPSDIAVEWESNGQPENNYKTTPP<br>VLDSDGSFFLYSKLTVDKSRWQQGNVFSCSVMHEALHNHYTQKS<br>LSLSPGK**LE***QVTLKESGPGILQPSQTLSLTCSFSGFSLSTFGMG*<br>*VGWIRQPSGKGLEWLAHIWWDDDKYYNPALKSRLTISKDTSENQ*<br>*VFLKIANVDTADTATYYCVRIAGTRYFDVWGTGTTVTVSS***RS*****ST***<br>***KGPKLEEGEFSEA*****QL***DIQMTQSPSSLSASLGGKVTITCKASQDI*<br>*NKFIAWYQHKPGKGPRLLIHYTSTLQPGIPSRFSGSGSGRDYSF*<br>*SISNLEPEDIATYYCLQYDNLYTFGGGTKLEIK*
31	Leader-Flag-scFvC40-CL	MNFGFSLIFLVLVLKGVQCEVKLVPR**QL**DYKDDDDK**EF***QVTLKE*<br>*SGPGILQPSQTLSLTCSFSGFSLSTFGMGVGWIRQPSGKGLEWL*<br>*AHIWWDDDKYYNPALKSRLTISKDTSENQVFLKIANVDTADTAT*<br>*YYCVRIAGTRYFDVWGTGTTVTVSS***RS*****STKGPKLEEGEFSEA*****QL**<br>*DIQMTQSPSSLSASLGGKVTITCKASQDINKFIAWYQHKPGKGP*<br>*RLLIHYTSTLQPGIPSRFSGSGSGRDYSFSISNLEPEDIATYYC*<br>*LQYDNLYTFGGGTKLEIK***GS**EIKRTVAAPSVFIFPPSDEQLKSG<br>TASVVCLLNNFYPREAKVQWKVDNALQSGNSQESVTEQDSKDST<br>YSLSSTLTLSKADYEKHKVYACEVTHQGLSSPVTKSFNRGEC

32	Leader-Flag-scFvC40-CH1/CH2/CH3(N297A)	MNFGFSLIFLVLVLKGVQCEVKLVPR**QL**DYKDDDDK**EF**QVTLKE<br>SGPGILQPSQTLSLTCSFSGFSLSTFGMGVGWIRQPSGKGLEWL<br>AHIWWDDDKYYNPALKSRLTISKDTSENQVFLKIANVDTADTAT<br>YYCVRIAGTRYFDVWGTGTTVTVSS**RSSTKGPKLEEGEFSEAQL**<br>DIQMTQSPSSLSASLGGKVTITCKASQDINKFIAWYQHKPGKGP<br>RLLIHYTSTLQPGIPSRFSGSGSGRDYSFSISNLEPEDIATYYC<br>LQYDNLYTFGGGTKLEIK**GS**SSASTKGPSVFPLAPSSKSTSGGT<br>AALGCLVKDYFPEPVTVSWNSGALTSGVHTFPAVLQSSGLYSLS<br>SVVTVPSSSLGTQTYICNVNHKPSNTKVDKKVEPKSCDKTHTCP<br>PCPAPELLGGPSVFLFPPKPKDTLMISRTPEVTCVVVDVSHEDP<br>EVKFNWYVDGVEVHNAKTKPREEQYASTYRVVSVLTVLHQDWLN<br>GKEYKCKVSNKALPAPIEKTISKAKGQPREPQVYTLPPSRDELT<br>KNQVSLTCLVKGFYPSDIAVEWESNGQPENNYKTTPPVLDSDGS<br>FFLYSKLTVDKSRWQQGNVFSCSVMHEALHNHYTQKSLSLSPGK
33	Leader-Flag-C40-IgG1(N297A)-TNC	MNFGFSLIFLVLVLKGVQCEVKLVPR**QL**DYKDDDDK**EF**QVTLKE<br>SGPGILQPSQTLSLTCSFSGFSLSTFGMGVGWIRQPSGKGLEWL<br>AHIWWDDDKYYNPALKSRLTISKDTSENQVFLKIANVDTADTAT<br>YYCVRIAGTRYFDVWGTGTTVTVSS**RS**SSASTKGPSVFPLAPSS<br>KSTSGGTAALGCLVKDYFPEPVTVSWNSGALTSGVHTFPAVLQS<br>SGLYSLSSVVTVPSSSLGTQTYICNVNHKPSNTKVDKKVEPKSC<br>DKTHTCPPCPAPELLGGPSVFLFPPKPKDTLMISRTPEVTCVVV<br>DVSHEDPEVKFNWYVDGVEVHNAKTKPREEQYASTYRVVSVLTV<br>LHQDWLNGKEYKCKVSNKALPAPIEKTISKAKGQPREPQVYTLP<br>PSRDELTKNQVSLTCLVKGFYPSDIAVEWESNGQPENNYKTTPP<br>VLDSDGSFFLYSKLTVDKSRWQQGNVFSCSVMHEALHNHYTQKS<br>LSLSPGK**LE**DIACGCAAAPDIKDLLSRLEELEGLVSSLREQGTG
34	Leader-Flag-C4-IgG1(N297A)-scFvC9	MNFGFSLIFLVLVLKGVQCEVKLVPR**QL**DYKDDDDK**EL**QVQLLQ<br>SGPELVKPGASVKLSCKASGYSFTSYDINWVKQRPGQGLEWVGW<br>IYPRDGDTKYNEKFKGKAILTVDTSSNTAYMNLHSLTSEDSAVY<br>FCARLTGPYWYFDVWGTGTTVTVSS**GS**SSASTKGPSVFPLAPSS<br>KSTSGGTAALGCLVKDYFPEPVTVSWNSGALTSGVHTFPAVLQS<br>SGLYSLSSVVTVPSSSLGTQTYICNVNHKPSNTKVDKKVEPKSC<br>DKTHTCPPCPAPELLGGPSVFLFPPKPKDTLMISRTPEVTCVVV<br>DVSHEDPEVKFNWYVDGVEVHNAKTKPREEQYASTYRVVSVLTV<br>LHQDWLNGKEYKCKVSNKALPAPIEKTISKAKGQPREPQVYTLP<br>PSRDELTKNQVSLTCLVKGFYPSDIAVEWESNGQPENNYKTTPP<br>VLDSDGSFFLYSKLTVDKSRWQQGNVFSCSVMHEALHNHYTQKS<br>LSLSPGK**EFLE**QIQLVQSGPELKKPGETVKISCKASGYTFTTAG<br>MQWVQKMPGKGFKWIGWINTHSGEPKYAEDFKGRFAFSLETSAS<br>TAYLQISNLKNEDTATYFCARWDGTGYWGQGTTLTVSS**RSSTKG**<br>**PKLEEGEFSEAQL**QIVLTQSPAIMSASPGEKVTITCSASSSVSY<br>MHWFQQKPGTSPKLWIYSTSNLASGVPARFSGSGSGTSYSLTIS<br>RMEAEDAATYYCQQRSSYPPTFGGGTKLEIK
35	Leader-Flag-C4-IgG1(N297A)-scFvC19	MNFGFSLIFLVLVLKGVQCEVKLVPR**QL**DYKDDDDK**EL**QVQLLQ<br>SGPELVKPGASVKLSCKASGYSFTSYDINWVKQRPGQGLEWVGW<br>IYPRDGDTKYNEKFKGKAILTVDTSSNTAYMNLHSLTSEDSAVY<br>FCARLTGPYWYFDVWGTGTTVTVSS**GS**SSASTKGPSVFPLAPSS<br>KSTSGGTAALGCLVKDYFPEPVTVSWNSGALTSGVHTFPAVLQS<br>SGLYSLSSVVTVPSSSLGTQTYICNVNHKPSNTKVDKKVEPKSC<br>DKTHTCPPCPAPELLGGPSVFLFPPKPKDTLMISRTPEVTCVVV<br>DVSHEDPEVKFNWYVDGVEVHNAKTKPREEQYASTYRVVSVLTV<br>LHQDWLNGKEYKCKVSNKALPAPIEKTISKAKGQPREPQVYTLP<br>PSRDELTKNQVSLTCLVKGFYPSDIAVEWESNGQPENNYKTTPP<br>VLDSDGSFFLYSKLTVDKSRWQQGNVFSCSVMHEALHNHYTQKS<br>LSLSPGK**EFLE**QIQLVQSGPEVKKPGETVKISCKASGYTFTIHG<br>MSWVKQAPGKGLKWMGWINTYSGVPTYANDFKGRFAFSLETSAS<br>TAYLQINNLKNEDTATYFCARDEVRRGFGFAYWGQGTLVTVSA**R**<br>**SSTKGPKLEEGEFSEAQL**DIVMAQSQKFMSVSVGDRVSVTCKAS<br>QNVGTNVAWYQQRPGQSPKALIYSASYRYSGVPDRFTGSGSGTD<br>FTLTISNVQSEDLAEYFCQQFDSHPLTFGAGTKLELK
36	Leader-Flag-C4-IgG1(N297A)-scFvC40	MNFGFSLIFLVLVLKGVQCEVKLVPR**QL**DYKDDDDK**EL**QVQLLQ<br>SGPELVKPGASVKLSCKASGYSFTSYDINWVKQRPGQGLEWVGW

		IYPRDGDTKYNEKFKGKAILTVDTSSNTAYMNLHSLTSEDSAVY<br>*FCARLTGPYWYFDVWGTGTTVTVSS***GS**SSASTKGPSVFPLAPSS<br>KSTSGGTAALGCLVKDYFPEPVTVSWNSGALTSGVHTFPAVLQS<br>SGLYSLSSVVTVPSSSLGTQTYICNVNHKPSNTKVDKKVEPKSC<br>DKTHTCPPCPAPELLGGPSVFLFPPKPKDTLMISRTPEVTCVVV<br>DVSHEDPEVKFNWYVDGVEVHNAKTKPREEQYASTYRVVSVLTV<br>LHQDWLNGKEYKCKVSNKALPAPIEKTISKAKGQPREPQVYTLP<br>PSRDELTKNQVSLTCLVKGFYPSDIAVEWESNGQPENNYKTTPP<br>VLDSDGSFFLYSKLTVDKSRWQQGNVFSCSVMHEALHNHYTQKS<br>LSLSPGK**EFLE***QVTLKESGPGILQPSQTLSLTCSFSGFSLSTFG*<br>*MGVGWIRQPSGKGLEWLAHIWWDDDKYYNPALKSRLTISKDTSE*<br>*NQVFLKIANVDTADTATYYCVRIAGTRYFDVWGTGTTVTVSS***RS**<br>***STKGPKLEEGEFSEA*****QL***DIQMTQSPSSLSASLGGKVTITCKASQ*<br>*DINKFIAWYQHKPGKGPRLLIHYTSTLQPGIPSRFSGSGSGRDY*<br>*SFSISNLEPEDIATYYCLQYDNLYTFGGGTKLEIK*
37	Leader-C4-LC-GpL<br>Flag less	MNFGFSLIFLVLVLKGVQCEVKLVPR**QL***DIVMTQSHKFMSTSVG*<br>*DRVSITCKASQDVDTAVAWYQQKPGQSPKLLIYWASTRHTGVPD*<br>*RFTGSGSGTDYTLTISSVQAEDLARYYCQQYYSVPPTFGGGTKL*<br>**GS**EIKRTVAAPSVFIFPPSDEQLKSGTASVVCLLNNFYPREAKV<br>QWKVDNALQSGNSQESVTEQDSKDSTYSLSSTLTLSKADYEKHK<br>VYACEVTHQGLSSPVTKSFNRGEC**LE**KPTENNEDFNIVAVASNF<br>ATTDLDADRGKLPGKKLPLEVLKEMEANARKAGCTRGCLICLSH<br>IKCTPKMKKFIPGRCHTYEGDKESAQGGIGEAIVDIPEIPGFKD<br>LEPMEQFIAQVDLCVDCTTGCLKGLANVQCSDLLKKWLPQRCAT<br>FASKIQGQVDKIKGAGGD
38	Leader-Flag-C4-<br>IgG1(N297A)	MNFGFSLIFLVLVLKGVQCEVKLVPR**QL**DYKDDDDK**EF***QVQLLQ*<br>*SGPELVKPGASVKLSCKASGYSFTSYDINWVKQRPGQGLEWVGW*<br>*IYPRDGDTKYNEKFKGKAILTVDTSSNTAYMNLHSLTSEDSAVY*<br>*FCARLTGPYWYFDVWGTGTTVTVSS***RS**SSASTKGPSVFPLAPSS<br>KSTSGGTAALGCLVKDYFPEPVTVSWNSGALTSGVHTFPAVLQS<br>SGLYSLSSVVTVPSSSLGTQTYICNVNHKPSNTKVDKKVEPKSC<br>DKTHTCPPCPAPELLGGPSVFLFPPKPKDTLMISRTPEVTCVVV<br>DVSHEDPEVKFNWYVDGVEVHNAKTKPREEQYASTYRVVSVLTV<br>LHQDWLNGKEYKCKVSNKALPAPIEKTISKAKGQPREPQVYTLP<br>PSRDELTKNQVSLTCLVKGFYPSDIAVEWESNGQPENNYKTTPP<br>VLDSDGSFFLYSKLTVDKSRWQQGNVFSCSVMHEALHNHYTQKS<br>LSLSPGK

Supplemental Table S3. Antibodies and variants thereof used in this study. For the amino acid sequences of the expression plasmid encoded proteins please see supplemental table SI.

Proteine name		Expression plasmid(s) no. in table SI	
Short	Systematic name		
C4-(1)	C4-Fab1-LC:scFvC4	1	2
C4-(2)	C4-Fab2-LC:scFvC4	1	3
C4-(3)	C4-IgG1(N297A)-HC:scFvC4	4	5
C4-(4)	C4-IgG1(N297A)-LC:scFvC4	1	38
C4-(5)	C4-IgG1(N297A)-LC:scFvC4-HC:scFvC4	1	5
C4-(6)	C4(scFv)-IgG1(N297A)-HC:scFvC4	6	7
C4-(7)	C4(scFv)-IgG1(N297A)-LC:scFvC4	8	9
C4-(8)	C4(scFv)-IgG1(N297A)-LC:scFvC4-HC:scFvC4	8	7
C4-(9)	C4-IgG1(N297A)-HC:TNC-scFvC4	4	10
C4-(10)	C4-Fab1	4	2
C4-(11)	C4-Fab2	4	3
C4-(12)	C4-IgG1(N297A)	4	38
C4-(13)	C4(scFv)-IgG1(N297A)	6	9
C4-(14)	C4-IgG1(N297A)-HC:TNC	4	11
C4-(15)	C4(scFv)-TNC-Fc(DANA)	12	
C4-(16)	C4(scFv)-Fc(DANA)-TNC	13	
C4-(17)	C4-IgG1(N297A)-HC:RGY	4	14
C4-(18)	C4(scFv)-IgG1(N297A)-HC:TNC	6	15
C9-(12)	C9-IgG1(N297A)	16	17
C9-(3)	C9-IgG1(N297A)-HC:scFvC9	16	18
C9-(13)	C9(scFv)-IgG1(N297A)	19	20
C9-(14)	C9-IgG1(N297A)-HC:TNC	16	21
C19-(12)	C19-IgG1(N297A)	22	23
C19-(3)	C19-IgG1(N297A)-HC:scFvC19	22	24
C19-(13)	C19(scFv)-IgG1(N297A)	25	26
C19-(14)	C19-IgG1(N297A)-HC:TNC	22	27
C40-(12)	C40-IgG1(N297A)	28	29
C40-(3)	C40-IgG1(N297A)-HC:scFvC40	28	30
C40-(13)	C40(scFv)-IgG1(N297A)	31	32
C40-(14)	C40-IgG1(N297A)-HC:TNC	28	33
C4/C9-(3)	C4-IgG1(N297A)-HC:scFvC9	4	34
C4/C19-(3)	C4-IgG1(N297A)-HC:scFvC19	4	35
C4/C40-(3)	C4-IgG1(N297A)-HC:scFvC40	4	36
C4-(3)-GpL	C4-IgG1(N297A)-HC:scFvC4-LC:GpL	37	5
C4-(12)-GpL	C4-IgG1(N297A)-LC:GpL	37	38
